# Method for predicting the wettability of micro-structured surfaces by continuum phase-field modelling

**DOI:** 10.1016/j.mex.2023.102458

**Published:** 2023-10-24

**Authors:** Marina Provenzano, Francesco Maria Bellussi, Matteo Morciano, Pietro Asinari, Matteo Fasano

**Affiliations:** a Department of Energy, Politecnico di Torino, Corso Duca degli Abruzzi 24, Torino, 10129, Italy; b Istituto Nazionale di Ricerca Metrologica, Strada delle Cacce 91, Torino, 10135, Italy

**Keywords:** Surface engineering, Diffuse-interface model, Phase-field model, Wettability, Additive manufacturing, Sessile droplet, Method for predicting the wettability of micro-structured surfaces by continuum phase-field modelling

## Abstract

Numerical prediction of material properties is attracting the attention of the scientific community and industry because of its usefulness in the design process. In the fields of fluid dynamics and microfluidics, several simulation methods have been proposed and adopted to evaluate the properties of surfaces and material interfaces, thanks to the increasing computational power available. However, despite the efforts made, a general and standardized methodology for implementing such methods is still lacking, thus requiring a trial-and-error approach for each new problem, making them difficult to implement and creating a bottleneck at the initial stage of surface design. Here, we report a validated protocol to evaluate the wettability of micro-structured surfaces with a phase-field model. Summarizing:

• Simulating physical phenomena with multi-phase flows and moving contact lines can be a challenging task, due to the coupling among disparate length scales.
• Using the Cahn-Hilliard diffuse-interface model, moving contact lines can be extensively investigated, although difficulties may arise when implementing numerical simulations, e.g., model parameter calibration, selection of boundary conditions, post-processing of fluid dynamics/equilibrium.
• A method for employing this model and evaluating the physical consistency of the results is proposed here, considering the wettability of micro-structured surfaces as a case study.

## Graphical abstract

**Figure:**
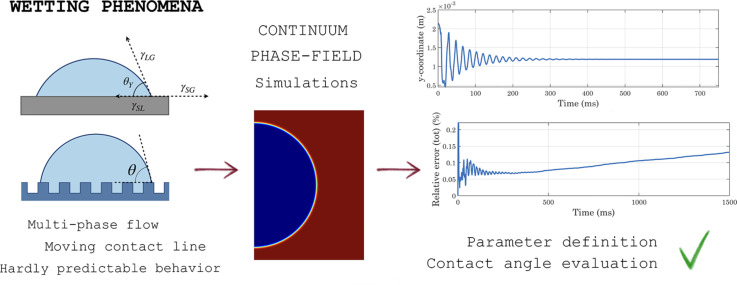

Specification table

**Table:** 

Subject area:	Engineering
More specific subject area:	Surface wetting
Name of your method:	Method for predicting the wettability of micro-structured surfaces by continuum phase-field modelling
Name and reference of original method:	Diffuse-interface model based on the Navier-Stokes and Cahn-Hilliard equations [1], [2], [3]
Resource availability:	Finite element modelling software

## Method Details

The work presented here is organized into the following sections:

1. *Theoretical background*, in which the theory of surface wetting phenomena is recalled.
2. *Modelling alternatives*, in which we review the modelling methods available in the literature.
3. *Theoretical background of boundary conditions*, in which the boundary conditions for multi-phase simulations are discussed.
4. *Dimensionless form of model equations*, in which we propose the dimensionless form of the governing equations adopted.
5. *Finite element method*, where we describe the formulation of the finite element method used in this work.
6. *Details on implemented boundary conditions*, in which we describe the chosen boundary conditions and their effects on result reliability.
7. *Simulation post-processing*, in which we describe the methods used to post-process numerical simulations and evaluate their physical consistency.
8. *Calibration of phase-field model parameters*, in which we identify the most important parameters to be defined in phase-field simulations and describe the calibration protocol to obtain meaningful physical results.

Readers who are already familiar with the theoretical background of wetting phenomena and multi-phase simulations may proceed directly to Section 4.

## Theoretical background

**Fig. 1:**
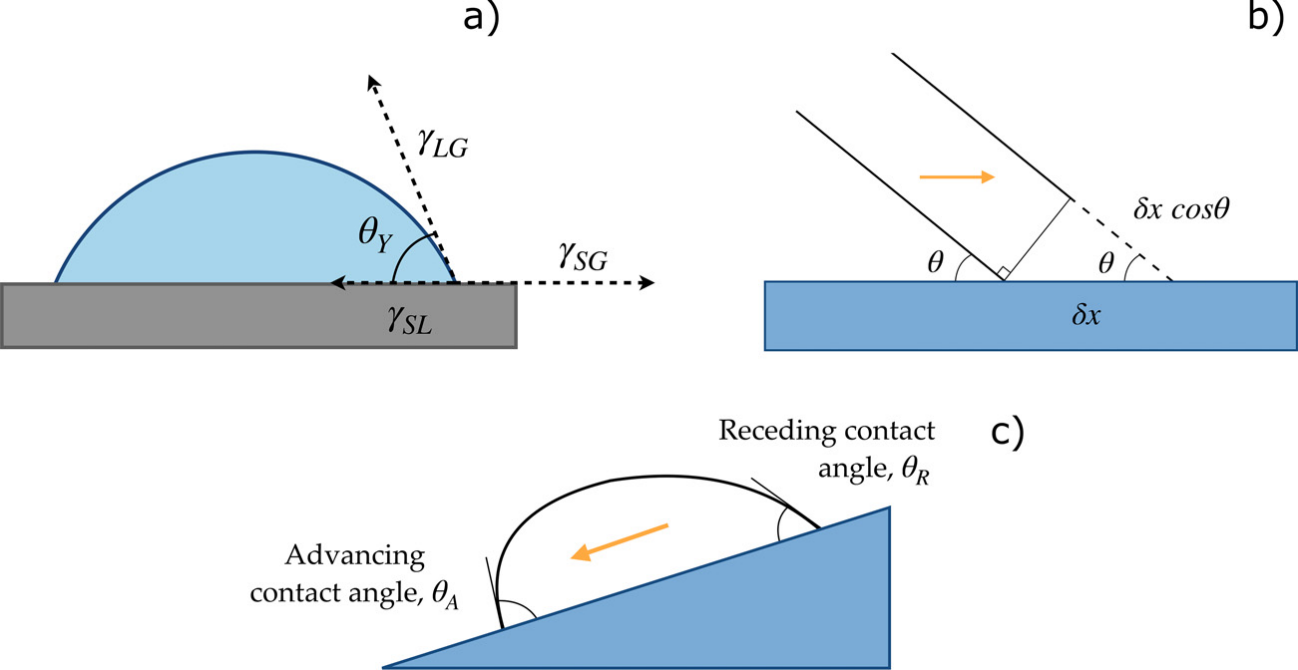
(a) Force balance at the contact line. (b) Displacement of the contact line on a solid smooth surface ($\theta =\theta_{Y}$). (c) Advancing and receding contact angles. The contact angle hysteresis (CAH) should be zero in the presence of an ideal, smooth, and homogeneous surface, whereas for real surfaces it is generally between 5${}^{\circ }$ and 20${}^{\circ }$[13], [14].

When a liquid is in contact with a solid surface in the presence of a third immiscible fluid phase (usually a gas), it can either create a film or generate a droplet, depending on the solid-liquid ($\gamma_{SL}$), solid-gas ($\gamma_{SG}$), and liquid-gas ($\gamma_{LG}$) interfacial surface tensions. The first scenario occurs when $S=\gamma_{SG}-\left(\gamma_{SL}+\gamma_{LG}\right)>0$; on the contrary, if S<0 the three phases meet along the three-phase contact line (TPL) [4]. The angle arising at the TPL, between the tangent to the liquid-gas interface and the solid surface, is known as the equilibrium contact angle $\theta_{Y}$, defined by Young [5] for the case of a perfectly smooth surface (Fig. 1a). The related surface free energy of the system can be generically described as follows: $E=\gamma_{SL}A_{SL}+\gamma_{SG}A_{SG}+\gamma_{LG}A_{LG}$, being $A_{SL}$ the solid-liquid interfacial area, $A_{SG}$ the solid-gas area and $A_{LG}$ the liquid-gas area [4]. If the contact line moves by an infinitesimal distance δx, the surface free energy undergoes a change $\delta E=\gamma_{SL}\delta A_{SL}-\gamma_{SG}\delta A_{SL}+\gamma_{LG}\delta A_{SL}\cos\left(\theta_{Y}\right)$, whereas the bulk energy remains constant (unless the volume of liquid changes, see Fig. 1b). When δx→0, the ratio $\frac{\delta E}{\delta A_{SL}}$ approaches zero, leading to Young’s equation [6]:

(1) $$\gamma_{SG}=\gamma_{SL}\;+\;\gamma_{LG}\;\cos\left(\theta_{Y}\right).$$

The liquid forms a spherical cap on a perfectly smooth solid surface with a contact angle (CA) equal to $\theta_{Y}$ when S<0 and neglecting gravity; the latter condition occurs if the characteristic size of the system is smaller than the capillary length, defined as: $l_{c}=\sqrt{\frac{\gamma_{LG}}{\rho g}}$ (ρ is the liquid density, g the gravitational acceleration) [7]. A surface is considered “phylic” with respect to the liquid it is interacting with if $\theta_{Y}<90^{\circ }$, “phobic” otherwise. However, these distinctions should not be applied restrictively, as changes in the wettability of a surface are actually gradual [8]. Eq. 1 is still widely used and accepted, although $\gamma_{SL}$ and $\gamma_{SG}$ are difficult to evaluate. Moreover, real surfaces are not atomically smooth but show microscopic imperfections, so the movement of droplets can be hindered. Therefore, two characteristic contact angles, namely the advancing $\theta_{A}$ and receding $\theta_{R}$ contact angles, can be defined. These can be measured by tilting the surface, as schematically represented in Fig. 1c. The force component parallel to the surface deforms the droplet before it begins to descend, and the difference between $\theta_{A}$ and $\theta_{R}$ is known as contact angle hysteresis (CAH) [9], [10]. When contact angle hysteresis is high, drops cannot be easily removed from the substrate, even at high contact angles, so it plays a key role in wettability. It should also be noted that for real surfaces Young’s contact angle is replaced by an apparent static CA (θ), which can be observed macroscopically and lies between $\theta_{R}$ and $\theta_{A}$. A rough or structured surface differs from a smooth surface as it exhibits local deviations from the ideal plane. Peaks and valleys may have different characteristic lengths and may be undesirable or created according to an appropriate geometric pattern (highly ordered structures) [11], [12].

**Fig. 2:**
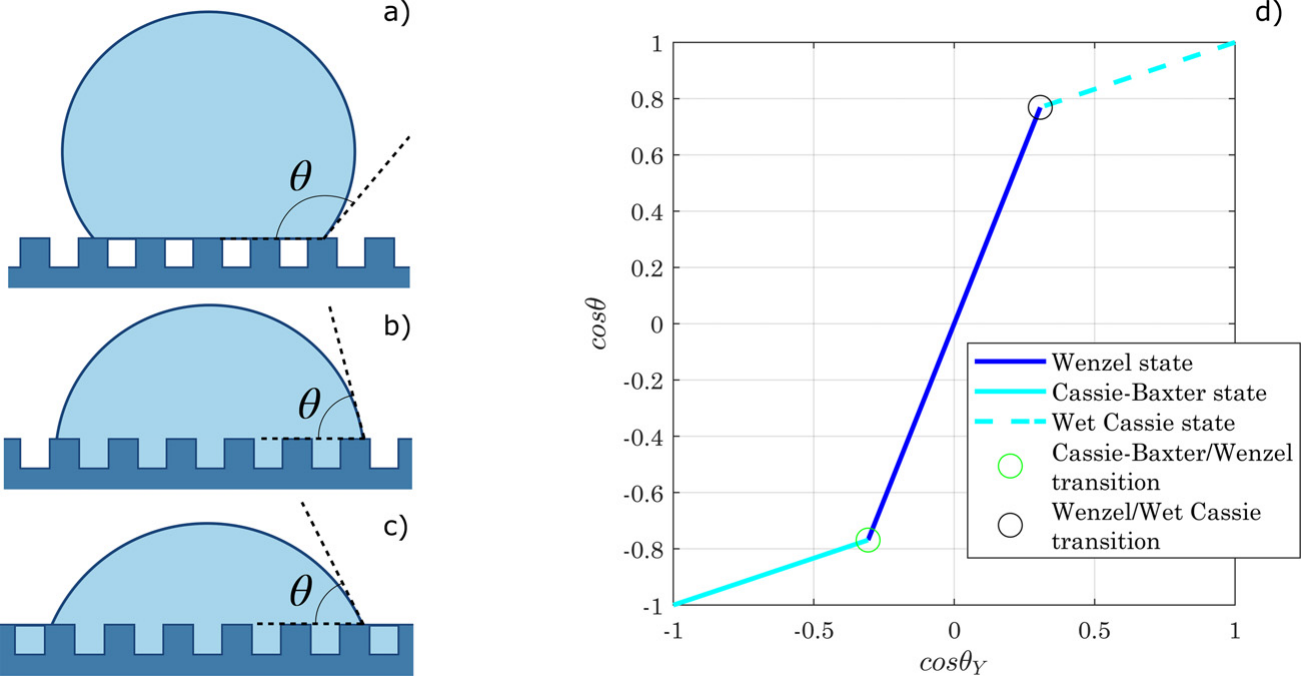
Schematic representation of wetting states on a structured solid surface, according to (a) Cassie-Baxter, (b) Wenzel, and (c) Wet Cassie models. (d) Relationship between the apparent contact angle on a rough surface (θ) and the Young contact angle of a perfectly smooth surface made of the same material ($\theta_{Y}$), for different wetting models ($r=2.5,\;\phi_{S}=0.3$).

The well-known Wenzel [15] and Cassie-Baxter [16] models are a commonly accepted guideline for studying the wettability of non-flat surfaces and for calculating their contact angle (Fig. 2). According to Wenzel’s model, suitable for both hydrophobic and hydrophilic materials, liquid penetrates the grooves of a rough surface, filling them completely while forming a finite macroscopic contact angle $\theta_{W}$. Due to roughness, the actual surface area is greater than the projected one: the wettability of the system depends on the interfacial tensions and the magnitude of the solid/liquid contact area. In detail, assuming that the contact line moves by an infinitesimal amount δx, the surface free energy changes as follows: $\delta E=\left(\gamma_{SL}-\gamma_{SG}\right)r\delta x+\gamma_{LG}\delta x\cos\theta$, being r the roughness factor $r=\frac{\text{Total}\;\text{surface}\;\text{area}}{\text{Projected}\;\text{surface}\;\text{area}}>1$ [6], [7]. If r=1 the surface is perfectly smooth and Young’s equation is recovered. If r>1:

(2) $$\cos\theta_{W}=r\cos\theta_{Y}.$$

In the Wenzel state, therefore, roughness amplifies the wetting trend of a smooth surface: a hydrophobic material has an apparent CA greater than Young’s one, whereas a hydrophilic material becomes more easily wettable. Eq. 2, however, can lead to non-physical results for high roughness factors (e.g., $\cos\theta_{W}>1$ or $\cos\theta_{W}<-1$). Lastly, due to liquid penetration within the surface roughness, the contact angle hysteresis for drops that are in a Wenzel state can be significant. In the Cassie-Baxter state, on the other hand, the liquid does not fill the grooves of a rough (or geometrically structured) surface, but there are air pockets trapped between the solid substrate and the droplet. The latter is therefore suspended, resulting in a superhydrophobic condition related to the “lotus effect”. Again, if the contact line moves along the solid substrate, the change in surface free energy can be evaluated as follows, where $\phi_{S}$ is the fraction of the droplet base in contact with the surface [6], [7]: $\delta E=\left(\gamma_{SL}-\gamma_{SG}\right)\phi_{S}\delta x+\left(1-\phi_{S}\right)\gamma_{LG}\delta x+\gamma_{LG}\delta x\cos\theta$. At equilibrium, when δx→0 also $\frac{\delta E}{\delta x}$ approaches zero:

(3) $$\cos\theta_{CB}=\phi_{S}-1+\phi_{S}\cos\theta_{Y}.$$

When $\phi_{S}\to 0$, $\theta_{CB}\to$ 180${}^{\circ }$. The same result can be obtained from a more general equation developed by Cassie [17], which describes the contact angle on a flat surface with a heterogeneous composition as a weighted average:

(4) $$\cos\theta =\sum_{i=1}^{N}\phi_{i}\cos\theta_{Y,i},$$

being $\phi_{i}$ the fraction of area with $\theta_{Y,i}$ as contact angle. If the surface consists of two different materials, one of them being air ($\theta_{1}=\theta_{Y}$, $\theta_{2}=180^{\circ }$), Eq. 3 emerges. In the Cassie-Baxter state, also known as the “fakir state”[7], contact angle hysteresis is reduced, and droplets can easily roll off the surface. For a certain material and surface structure, the configuration involving the least change in surface energy for an infinitesimal displacement of the TPL prevails. The transition condition from a Wenzel to a Cassie-Baxter state is obtained, therefore, by imposing $\delta E_{W}>\delta E_{CB}$: $\cos\theta_{Y}<\frac{\phi_{S}-1}{r-\phi_{S}}<0$. It seems clear that the Cassie-Baxter state can emerge as a global energy minimum condition only with intrinsically hydrophobic materials, although metastable configurations may exist due to the presence of energy barriers opposing the Cassie-to-Wenzel wetting transition (their role in wettability has been the subject of several studies [18], [19]). Wenzel’s law is not applicable with low $\theta_{Y}$ values, at which hemi-wicking occurs and the so-called “Wet Cassie” state emerges: a certain amount of fluid invades the texture grooves, and the drop lies on a patchwork of liquid and solid [20]. Assuming that the liquid fills the gaps of the surface pattern, leaving the top of the structures dry, an infinitesimal displacement of the contact line would cause an energy change equal to $\delta E=\left(\gamma_{SL}-\gamma_{SG}\right)\left(r-\phi_{S}\right)\delta x+\gamma_{LG}\left(1-\phi_{S}\right)\delta x$. If δE<0, the liquid would spread indefinitely on the surface, which leads to the following condition: $\cos\theta_{Y}>\frac{1-\phi_{S}}{r-\phi_{S}}>0$. At this point, Eq. 4 can be applied with the following impositions, $\theta_{1}=\theta_{Y},\;\theta_{2}=$ 0${}^{\circ },\;\phi_{1}=\phi_{S},\;\phi_{2}=1-\phi_{S}$, thus obtaining a descriptive equation for the Wet Cassie state:

(5) $$\cos\theta_{CW}=1-\phi_{S}+\phi_{S}\cos\theta_{Y}.$$

The transition criterion can be also achieved by equating Eqs. 2 and 5.

Although these models are still widely used as references in the study of wettability, they have shown limits for describing all behaviors found in nature [21], [22], [23]. According to Patankar’s research [24], for example, there are multiple equilibrium states for a droplet on a rough surface corresponding to local energy minima, and the actual shape taken by the drop depends on how it was formed.

## Modelling alternatives

Several modelling approaches are currently used to study the dynamics of wetting phenomena and simulate the impact and propagation of a droplet on a surface under the continuum assumption [25]. The classical hydrodynamic model, for example, uses the Navier-Stokes equations (momentum balance and mass conservation), which for an incompressible, chemically homogeneous, non-reactive Newtonian fluid with no electrical charges can be written as [7]:

(6) $$\rho (\frac{\partial u}{\partial t}+u\cdot \nabla u)=F-\nabla \cdot T$$

(7) ∇·u=0,

where u is the velocity field, t the time, F represents a body force (e.g., the gravity force: F=ρg, where ρ is the density and g the gravitational acceleration) and $T=pI-\mu \left[\nabla u+{\left(\nabla u\right)}^{T}\right]+\mu (\frac{2}{3}\nabla \cdot u)I$ is the stress tensor (p is the pressure, μ the dynamic viscosity, I the identity matrix, and $E=\frac{1}{2}\left[\nabla u+{\left(\nabla u\right)}^{T}\right]$ represents the deviatoric stress tensor). In this approach, the liquid-gas interface constitutes a domain boundary and the surface tension $\gamma_{LG}$ is assumed as a boundary condition without appearing explicitly in the Navier-Stokes equations. In the case of a free surface without surfactant adsorption or temperature gradients along the solid substrate, the interfacial tension gradients are negligible and the boundary conditions (BC) can be expressed as [4], [7]:

(8) $$\Delta p+\tau_{nn}{{|}_{ambient}-\tau_{nn}|}_{liquid}-\gamma_{LG}C=0$$

(9) $$\tau_{nt}{{|}_{ambient}-\tau_{nt}|}_{liquid}=0.$$

Eq.s 8 and 9 represent the stress balance at a free surface: $\tau_{nn}=n\cdot \tau \cdot n$ and $\tau_{nt}=n\cdot \tau \cdot t$ are the normal and the shear stress, respectively, $\tau =\mu [\nabla u+{\left(\nabla u\right)}^{T}]$ is the viscous stress tensor, $C=(\frac{1}{R_{1}}+\frac{1}{R_{2}})$ is the local mean curvature of the surface, n the unit normal of the liquid-gas interface, t the unit tangent vector. At equilibrium, Eq. 8 reduces to the Young-Laplace equation [6]:

(10) $$\Delta p=\gamma_{LG}(\frac{1}{R_{1}}+\frac{1}{R_{2}}),$$

where Δp denotes the pressure difference between the liquid mass and the gas. A numerical evaluation of the contact line motion using these equations thus requires a non-fixed mesh that can follow the deformation of the droplet, the liquid-gas interface being a domain boundary on which the aforementioned dynamic BC must be imposed. In addition, the chosen mesh must respect a kinematic condition at the interface:

(11) $$(u_{mesh}-u)\cdot n=0,$$

where $u_{mesh}$ is the velocity of the mesh along the liquid-gas interface. Moreover, coupling this model with a no-slip condition at the solid-fluid interface results in the emergence of a stress singularity at the three-phase contact line (see Section 3).

In the hydrodynamic model some additional information must be added to properly predict the evolution of the separation interface: relating the velocity of the contact line (TPL) to the contact angle (CA, θ) is the most widely used approach, although the phenomena occurring at the TPL are not fully explained yet. For instance, Cox’s theory assumes that θ is determined by viscous forces acting at the interface on a macroscopic length scale, whereas the equilibrium contact angle $\theta_{S}$ is preserved within the slip zone ($r\leq l_{s}$) [3], namely:

(12) $$g\left(\theta \right)=g\left(\theta_{S}\right)+Ca\ln(\frac{D}{l_{s}}),$$

where g is an algebraically complex function, D is the macroscopic length scale, $l_{s}$ the slip length (i.e., the artificial depth within the solid for which the velocity would reach zero [26]), and Ca the capillary number (i.e., the ratio of viscous forces to surface tension). In some cases Eq. 12 cannot accurately predict θ, so some corrections have been proposed, such as replacing the static contact angle, $\theta_{S}$, with a microscopic dynamic CA, $\theta_{D}$, related to the contact line velocity:

(13) $$\cos\theta_{S}-\cos\theta_{D}\left(Ca\right)=B{\left(Ca\right)}^{\zeta },$$

being B and ζ evaluated through a fitting procedure. The molecular-kinetic theory, on the other hand, correlates the contact line motion with the dynamics of molecules in close proximity to it: the deviation of the dynamic contact angle from the static value results in a capillary force enabling the molecules to move. This theory, however, does not take into account any viscous stress nor the geometric configuration of the surface, so it has no universal validity [4], [27]. Alternatively, the Shikhmurzaev model can be used, which introduces a surface tension gradient near the contact line. However, it requires phenomenological constants that cannot be determined *a priori*.

Multiphase flows and interface problems can alternatively be studied with interface-capturing methods, where auxiliary fields are used to describe interface behaviors (e.g., level-set, volume of fluid and phase-field methods). These models often do not require the use of moving meshes (as is the case of interface-tracking methods); however, they typically involve higher computational costs, issues related to the use of a finite interface thickness, and mass variations [4], [27], [28]. The level-set method (LS), for example, uses a fixed grid (e.g., Cartesian) to solve numerically the Navier-Stokes equations, and an advection algorithm enables the contact line motion. The interface is represented by the zero level set of a scalar smooth function $\phi_{LS}\left(x\right):R^{D}\to R,\;\Gamma =\left\{x:\phi_{LS}\left(x\right)=0\right\}$ [28]. The interface moves by convection due to the velocity field, which in turn is obtained as a solution of the momentum equation [29]:

(14) $$\frac{\partial \phi_{LS}}{\partial t}+\nabla \cdot \left(u\phi_{LS}\right)=0.$$

The LS method allows interface deformations and topology changes to be handled in a way that is fairly simple to implement, especially in its original formulation. However, it shows significant non-physical mass losses for large surface deformations. The method has been modified and enhanced over the years to improve mass conservation while preserving the original simplicity, resulting in the conservative level-set method (although its accuracy is reduced) [29], [30]. The volume of fluid method (VOF), as well as the previous one, can be used to study movements and deformations of the interface by means of a fixed computational grid, adopting for this purpose a smooth function $\phi_{VOF}$ which indicates the fraction of fluid present in each cell of the mesh. $\phi_{VOF}=1$ in one of the two phases (usually the liquid one) and $\phi_{VOF}=0$ for a cell filled with the second phase, while the interface is associated with $\phi_{VOF}=0.5$. The deformation of the liquid-gas surface is governed by an equation analogous to Eq. 14 [4], [28]:

(15) $$\frac{\partial \phi_{VOF}}{\partial t}+\nabla \cdot \left(u\phi_{VOF}\right)=0.$$

VOF methods suffer from less mass loss than LS methods, but there are still drawbacks related to the use of diffusive and unstable computational schemes [4], [28].

**Fig. 3:**
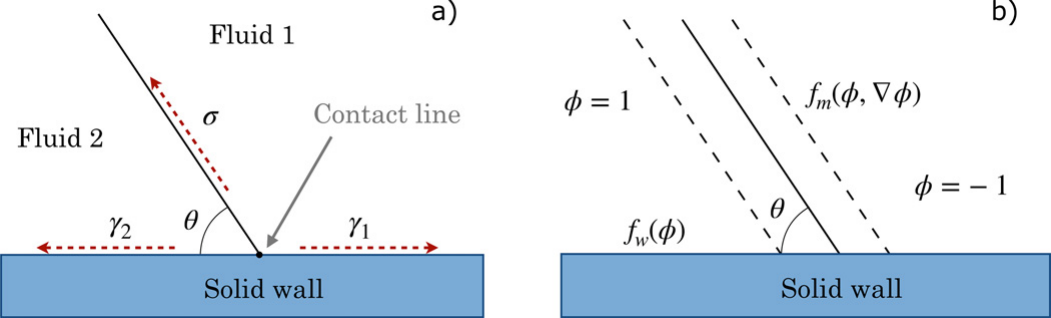
Schematic representation of the fluid-fluid separation interface according to (a) the hydrodynamic approach and (b) the phase-field model. Diffuse-interface methods replace the sharp interface with a transition region of finite thickness, through which the physical properties change rapidly but continuously.

In diffuse-interface models (DIM), the sharp interface (separating two different phases in the hydrodynamic approach) is replaced with a finite-thickness transition region (see Fig. 3), as is the case in phase-field methods. In this work, a phase-field model (PF) based on the Cahn-Hilliard equations [1], [31] was selected. Phase-field methods indeed provide results for multi-phase problems that exhibit good agreement with sharp-interface approaches, as reported in the benchmark study conducted by Aland and Voigt [32]. Further efforts have been dedicated to the study of binary fluids using phase-field methods, aiming to approach the sharp-interface limit by carefully selecting model parameters, such as interface thickness and interface mobility [2], [33]. Such a model allows the use of a fixed mesh and a no-slip condition at the solid-fluid interface: as also explained in Section 3, the phase-field model avoids the occurrence of the contact line paradox typically found in sharp-interface models, since the non-equilibrium of the chemical potential (and the resulting diffusion in a thin interface) leads to the TPL motion [34]. The governing equation is as follows [35]:

(16) $$\frac{\partial \phi }{\partial t}+u\cdot \nabla \phi =\nabla \cdot \left(M\nabla G\right),$$

where ϕ is the phase-field variable (ϕ∈[−1,1]), $M=\chi \epsilon^{2}$ is the mobility parameter, χ is a tuning parameter, ϵ scales with the thickness of the interface, u is the velocity field, G is the chemical potential [2]. This advection-diffusion equation can be considered a generalization of Fick’s law since the flux is related to the gradient of the chemical potential through the phenomenological parameter M. The presence of the interface is taken into account by modifying the Navier-Stokes equations:

(17) $$\rho (\frac{\partial u}{\partial t}+u\cdot \nabla u)=-\nabla p+\nabla \cdot \mu \left[\nabla u+{\left(\nabla u\right)}^{T}\right]+G\nabla \phi +F.$$

Density ρ and viscosity μ are expressed as [35]: $\rho =\frac{1}{2}\left[\left(1-\phi \right)\rho_{1}+\left(1+\phi \right)\rho_{2}\right];\;\mu =\frac{1}{2}\left[\left(1-\phi \right)\mu_{1}+\left(1+\phi \right)\mu_{2}\right]$. Similarly, volume fractions are $V_{f1}=\frac{1-\phi }{2}$ and $V_{f2}=\frac{1+\phi }{2}$ [27], and as a result: $\rho =\rho_{1}V_{f1}+\rho_{2}V_{f2};\;\mu =\mu_{1}V_{f1}+\mu_{2}V_{f2}$. Subscripts 1 and 2 identify the two phases, respectively: we refer to the air as “Fluid 1” and to the water phase as “Fluid 2”. Further details are provided by the authors in the recently published research article [36].

The approaches introduced and discussed so far were all based on the *continuum assumption*, which is the one also considered in this work.

## Theoretical background on boundary conditions

When a viscous fluid is close to a surface, adherence to the wall is generally assumed, meaning that the fluid has the same velocity as the domain boundary (no-slip condition). This assumption gives rise to the following expression:

(18) $$(u-u_{w})\cdot t=0,$$

where t is a general unit vector tangent to the wall. However, this condition is only an approximation of more complex phenomena, and therefore it is not universally valid. For example, the no-slip condition cannot be applied to phenomena with Knudsen (Kn) numbers close to the unit, where $Kn=\frac{\lambda_{m}}{D}$ ($\lambda_{m}$ is the molecular mean free path length, whereas D is the length scale of the system). The no-slip condition is also a problem where a moving interface is in contact with a solid surface, as in wetting phenomena: under these circumstances, as mentioned above, the adoption of a classical hydrodynamic model in conjunction with this condition results in the emergence of a stress singularity at the TPL. The choice of a phase-field model in this study allowed us to overcome this limitation and use a no-slip condition for this analysis [36] because the motion of the contact line is guaranteed by the diffusive term of the Cahn-Hilliard equation (Eq. 16) even in the presence of a zero velocity field. The incongruity that emerges from adopting a no-slip condition with a moving contact line is not, however, a purely mathematical problem, although molecular studies allow the coexistence of the two to be explained [37]. Other studies, conversely, have shown a relative fluid-solid slip in some cases, and none of the models introduced to explain this phenomenon have been able to quantitatively assess the magnitude of this effect near the TPL [38].

The Navier BC (NBC) represents one of the slip models that can be used to relax the no-slip condition and avoid stress singularities. This BC assumes that the relative slip is proportional to the tangential viscous stress [39]. For a Newtonian fluid: $\xi \left(u-u_{w}\right){{|}_{t}+\left[\mu \left(\nabla u+{\left(\nabla u\right)}^{T}\right)\cdot n\right]|}_{t}=0$, where $\xi =\frac{\mu }{l_{s}}$ is the slip coefficient and $l_{s}$ the slip length. ξ is generally high, so the no-slip condition is an adequate approximation in most cases [40]. For a two-dimensional system with zero wall velocity and a non-penetration condition (namely, $(u-u_{w})\cdot n=0$, being n the outward-pointing normal unit vector), the previous equation becomes [4]: $u=l_{s}\frac{\partial u}{\partial y}$. The NBC, however, showed reliability only far from the contact line, whereas near the TPL the condition fails. Moreover, this approach makes it necessary – for multiphase flows – to use an additional condition that explicitly evaluates the contact angle [39]. Over the years, hybrid models combining the results of MD simulations with continuum approaches have been created, to overcome these limitations. One possible solution is to use the Cahn-Hilliard-Navier-Stokes equations together with Generalized Navier Boundary Conditions (GNBC), which includes no-slip and Navier BCs as approximations. According to this BC, valid only with Ca<0.1 [41], the slip velocity at the wall is proportional to the sum of a viscous component and an unbalanced Young’s stress, due to the interface deviations from the equilibrium configuration. With respect to the NBC, therefore, there is an additional term that vanishes if the microscopic dynamic contact angle $\theta_{D}$ becomes equal to the static value $\theta_{S}$. Unbalanced Young’s stress is expressed by [42]: $\int_{int}\tau_{Young}dx=\sigma \left(\cos\theta_{S}-\cos\theta_{D}\right)$, where $\sigma =\gamma_{LG}$ is the surface tension. Integration is performed through the interface, parallel to the wall.

The adoption of a phase-field model also implies the introduction of an auxiliary variable ϕ, thus requiring appropriate boundary conditions. In a system consisting of two fluids in contact with each other and with a solid surface, the free energy has an additional term $f_{w}$, due to the wall energy [3], [43]. Therefore, for a domain Ω: $F=\int_{\Omega }f_{m}\left(\phi ,\nabla \phi \right)d\Omega +\int_{\partial \Omega }f_{w}\left(\phi \right)dA$, $f_{w}\left(\phi \right)=-\sigma \cos\theta_{S}\frac{\phi (3-\phi^{2})}{4}+\frac{\gamma_{1}+\gamma_{2}}{2}$, where $f_{m}$ is the free fluid-fluid mixing energy density, ∂Ω is the solid surface (dA: infinitesimal surface element), σ is the fluid-fluid interfacial tension, $\gamma_{1}$ and $\gamma_{2}$ are the fluid-solid interfacial tensions. Given the Young equation, $f_{w}\left(\pm 1\right)=\gamma_{1,2}$. The surface chemical potential L is obtained by applying a variational procedure: $L=\lambda n\cdot \nabla \phi +f_{w}^{\prime }\left(\phi \right)$. The naturally resulting boundary condition is L=0, which corresponds to imposing a dynamic microscopic contact angle equal to the static equilibrium value [27], [44]:

(19) $$\cos\theta_{S}=\frac{n\cdot \nabla \phi }{\left|\nabla \phi \right|}.$$

The generalization of this condition takes into account non-equilibrium states, enabling during the flow the occurrence of a dynamic microscopic contact angle $\theta_{D}$ different from the static contact angle $\theta_{S}$ [3]:

(20) $$\frac{\partial \phi }{\partial t}+u\cdot \nabla \phi =-\Gamma L\left(\phi \right),$$

where Γ is a rate constant that, as also M, can be seen as a property of the material. However, the values of these parameters are often not known, so they are mostly treated as phenomenological parameters, constituting the following dimensionless groups: $S=\frac{\sqrt{\mu M}}{D}$ and $\Pi =\frac{1}{\mu \Gamma D}$. $l_{d}=\sqrt{\mu M}$ is the diffusion length, closely related to the slip length $l_{s}$ [2]. If the flow is sufficiently slow that the ϕ profile across the interface can be assumed to be the equilibrium profile, applying the previous equation at the contact line (ϕ=0) leads to: $\frac{\cos\theta_{S}-\cos\theta_{D}}{\sin\theta_{D}}=(\frac{2\sqrt{2}}{3}\frac{\Pi }{Cn})Ca$, where $Cn=\frac{\epsilon }{D}$ is the Cahn number, and Ca is the capillary number. If the velocity is low or Γ is high, the discrepancy between the microscopic dynamic contact angle and the static value $\theta_{S}$ is negligible, as long as the flow is slow enough not to distort the ϕ profile. Γ and M govern the interface behavior in the near-wall and outer region, respectively, so if properly modified they can compensate for each other’s effects. The limitations caused by the small values of $l_{s}$ and $l_{d}$ in real phenomena can therefore be overcome by developing appropriate computational strategies: the adoption of an artificially large S in performing calculations can in fact be compensated for by acting on Π as if it were an adjustable parameter, achieving the correct result on a macroscopic length scale. This condition can also be coupled with the Generalized Navier Boundary Condition [35]: $\xi \left(u-u_{w}\right)+\mu \left[\nabla u+{\left(\nabla u\right)}^{T}\right]\cdot n=L\left(\phi \right)\nabla \phi$, where the last term is the unbalanced Young’s stress. Under appropriate assumptions (and specifically if the relaxation parameter Γ of Eq. 20 tends to infinity [34]), the NBC can then be derived from the GNBC, and further reduced to the no-slip condition when the slip length goes to zero. The Cahn-Hilliard model thus allows the contact line problem to be studied in such a way as to include both viscous actions at the interface (analyzed by Cox, see Section 2) and local effects (studied by molecular-kinetic models). The main limitation in using this condition, besides the computational costs, is that adequately compensating for the use of high diffusion lengths would require knowing the exact values of Γ and M, which are generally unknown, or alternatively performing a fitting on experimental data [3].

## Dimensionless form of model equations

The equations governing the displacement of a droplet on a solid surface can be expressed in a dimensionless form, allowing a better understanding of the phenomenon and the identification of characteristic dimensionless numbers. A phase-field model based on the Cahn-Hilliard/Navier-Stokes equations ((7), (16) and 17) was chosen for this study, so the following non-dimensional variables can be used: $u^{*}=\frac{u}{U};\;x^{*}=\frac{x}{D};\;g^{*}=\frac{g}{g};\;t^{*}=\frac{tU}{D};\;p^{*}=\frac{p}{\rho_{ref}U^{2}};\;\rho^{*}=\frac{\rho }{\rho_{ref}};\;\mu^{*}=\frac{\mu }{\mu_{ref}}$. D, U, $\rho_{ref}$, $\mu_{ref}$ are reference quantities, which should be chosen appropriately according to the system studied. Substituting these definitions into the Cahn-Hilliard/Navier-Stokes equations gives:

(21) $$\rho^{*}(\frac{\partial u^{*}}{\partial t^{*}}+u^{*}\cdot \nabla^{*}u^{*})=-\nabla^{*}p^{*}+\frac{1}{Re}\nabla^{*}\cdot \mu^{*}\left[\nabla^{*}u^{*}+{\left(\nabla^{*}u^{*}\right)}^{T}\right]+\frac{1}{Fr^{2}}\rho^{*}g^{*}+\frac{3}{2\sqrt{2}}\frac{1}{We\cdot Cn}\left[-Cn^{2}\nabla^{*}{}^{2}\phi +\phi \left(\phi^{2}-1\right)\right]\nabla^{*}\phi$$

(22) $$\nabla^{*}\cdot u^{*}=0$$

(23) $$\frac{\partial \phi }{\partial t^{*}}+u^{*}\cdot \nabla^{*}\phi =\frac{3}{2\sqrt{2}}\frac{1}{Pe}\nabla^{*}{}^{2}\left[-Cn^{2}\nabla^{*}{}^{2}\phi +\phi \left(\phi^{2}-1\right)\right],$$

where $Re=\frac{\rho_{ref}UD}{\mu_{ref}}$ is the Reynolds number, the ratio of inertial to viscous forces; $Cn=\frac{\epsilon }{D}$ is the Cahn number, namely the ratio of interface thickness to characteristic length; $Pe=\frac{DU\epsilon }{M\sigma }$ is the Péclet number, the ratio of advection to diffusion (it differs from the classically used one since it is defined specifically for this model); $Fr^{2}=\frac{U^{2}}{gD}$ is the Froude number, the ratio of flow inertia to gravity effect; $We=\frac{\rho_{ref}U^{2}D}{\sigma }=CaRe$ is the Weber number, the ratio of inertial forces to surface tension; Ca is the capillary number, the ratio of viscous forces to surface tension.

## Finite element method

For the development of this work, the cross-platform COMSOL Multiphysics® software was used [45], which implements algorithms based on finite element methods (FEM) for solving the partial differential equations (PDEs) related to the problem under analysis. Finite element methods use the variational, or weak, formulation of the boundary value problem, which for simplicity is considered time-independent [6]:

(24) Du(x)=fonΩ

(25) $$R(u\left(x\right),\partial_{\alpha }u\left(x\right))=0\;on\;\partial \Omega ,$$

being Ω the computational domain, D a differential operator, $\partial_{\alpha }u\left(x\right)$ a space derivative and $R(u\left(x\right),\partial_{\alpha }u\left(x\right))$ a general set of BCs.

Let V be a space consisting of “admissible functions” $v:\overset{︸}{\Omega }\to R$, also called shape functions, defined on $\overset{︸}{\Omega }=\Omega \cup \partial \Omega$ and satisfying certain regularity requirements (the definition of which is beyond the scope of this analysis). Moreover, let B⊂V be a basis of V, that is, each element v∈V can be written as a linear combination of functions ϕ∈B. The weak solution u to problem 24-25 belongs to V and satisfies the following condition:

(26) $$\int_{\Omega }Du\left(x\right)v\left(x\right)dx=\int_{\Omega }fv\left(x\right)dx\;\;\;\;\;\forall v\in V$$

where

(27) $$v\left(x\right)=\sum_{n\in N}a_{n}\phi_{n}\left(x\right);\;\;u\left(x\right)=\sum_{i\in N}u_{i}\phi_{i}\left(x\right);\;\phi_{i,n}\in B.$$

The variational formulation of the problem makes it possible to deal with more general cases than the strong one: the two are equivalent if certain assumptions concerning the regularity of the functions involved are respected. The problem can be approximated by replacing V with a subset $V_{h}\subset V$, a finite dimensional space (i.e., containing only a finite number of mutually independent functions), and its components $v_{h}$ are definable using a finite number of parameters (degrees of freedom). The solution $u_{h}$ of the discrete problem will be an approximation of u. The finite element method provides a simple way to define the $V_{h}$ spaces used in the discrete variational formulation. The domain $\overset{︸}{\Omega }$ is subdivided into polygons/polyhedrons, the size of which can be chosen according to the desired degree of accuracy and computational time, and the basis functions are chosen to be polynomial. Each of these polynomial functions has a domain located on a small number of mesh elements, so the overlap between different functions is low. To modify the accuracy of the simulation, actions can be taken either on the size of the mesh elements or on the degree of the interpolating polynomials. Recalling that the solution $u_{h}$ is a linear combination of basis functions $u_{h}\left(x\right)=\sum_{i=1}^{N}u_{i}\phi_{i}\left(x\right),\;\phi_{i}\in B_{h}$, and using the linearity of the integral and derivative operators, a boundary value problem can be transformed into a system of linear algebraic equations:

(28) Au=f,

where $u\in R^{N}$ is the solution vector, $f\in R^{N}$ contains information about the forcing terms, and $A\in R^{NxN}$ is a sparse matrix, as a result of using spatially localized basis functions.

## Details on implemented boundary conditions

**Fig. 4:**
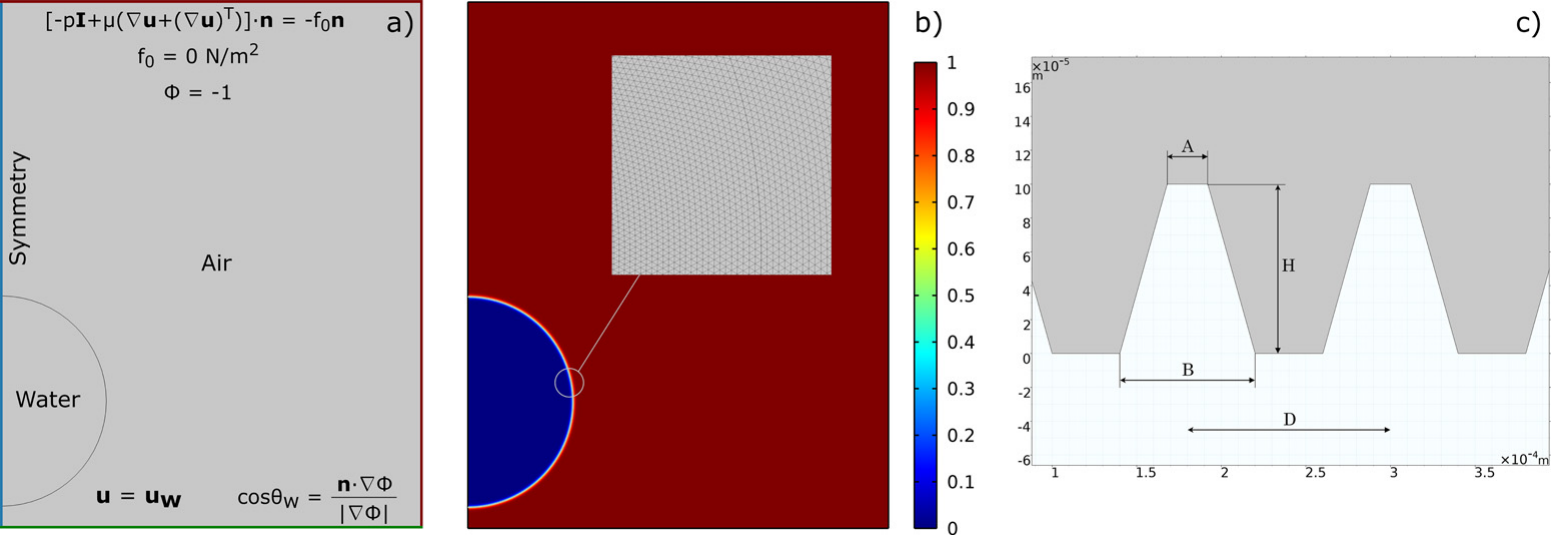
(a) Computational domain and boundary conditions used. (b) Simulation domain at the initial condition. The color bar indicates the air volume fraction. (c) Geometry of the micro-structured surface with details of the characteristic dimensions. As can be seen, a trapezoidal geometry was chosen. Figure taken from reference [36] and modified under license CC BY 4.0.

**Fig. 5:**
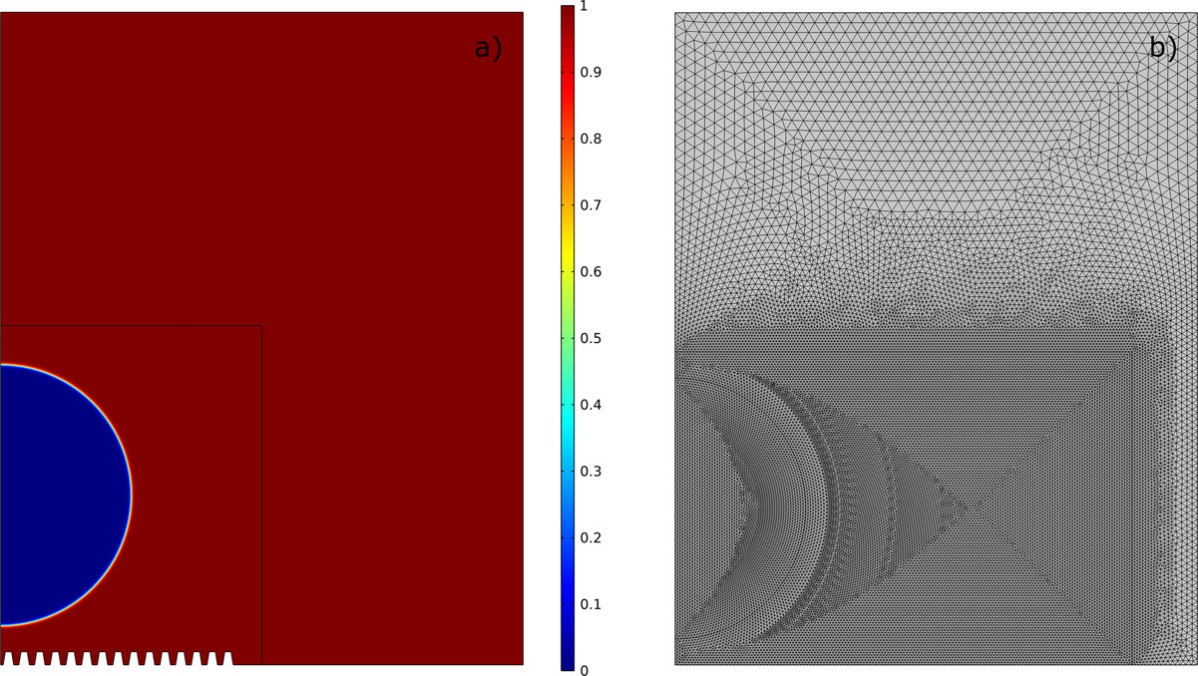
(a) Example of a simulation carried out with micro-structured surfaces, computational domain at the initial condition. The color bar indicates the air volume fraction. (b) Example of mesh used in simulations with perfectly smooth surfaces.

The scenario chosen for simulations consists of a droplet placed a few tenths of a millimeter from a solid surface, which settles on the surface after a short transient phase due to the action of gravity. Initially, perfectly smooth surfaces were considered to assess the physical reliability of the results in a simple case (Fig. 4). Next, some micro-structures were added to the computational domain (Fig. 5) [36].

The system of Cahn-Hilliard/Navier-Stokes equations considers the velocity field u=(u,v,w), the pressure p, and the phase ϕ as variables, which must then be defined properly along the boundaries of the chosen computational domain (see Fig. 4).

As for the solid-fluid wall, a no-slip condition (Eq. 18) coupled with the non-penetration condition was chosen. Moreover, the presence of zero diffusive flux across the wall was added:

(29) n·∇G=0,

combined with Eq. 19. Further details about these choices can be found elsewhere [36]. For the upper and right edges, open boundary conditions were applied to simulate an infinite domain:

(30) $$\left[-pI+\mu \left(\nabla u+{\left(\nabla u\right)}^{T}\right)\right]\cdot n=-f_{0}n;\;\;\;f_{0}=0\frac{N}{m^{2}}.$$

Regarding the value of the phase variable along these edges, as well as the conditions to be used on the left side, different configurations were compared:

• *2D, inlet/inlet*. A symmetry condition was imposed on the axis (i.e., the left edge of the domain), expressed by:
  (31) $$u\cdot n=0;\;\;\;K-\left(K\cdot n\right)n=0;\;\;\;K=[\mu \left(\nabla u+{\left(\nabla u\right)}^{T}\right)]\cdot n.$$
  Similarly, the flow of fluid phases across this edge was also set to zero. On the upper and right edges, on the other hand, the phase function was defined as
  (32) ϕ=−1.
• *2D, inlet/outlet*. The boundary conditions are the same as in the previous point, but an outlet condition was imposed on the right side, corresponding to a net outflow from the domain.
• *2D, outlet/outlet*. The outlet condition was chosen for both the upper and right edges.
• *3D, inlet/inlet*. In this case, an axisymmetry condition was applied on the axis, thus simulating a three-dimensional domain.

In all of these cases, the simulations resulted in a final contact angle comparable to that imposed on the solid wall, so this parameter was not used as a benchmark to evaluate the quality of the numerical results. Therefore, we checked the conservation of mass: there are no reactions in the system, so the total mass of water shall be constant. The water mass per unit volume can be defined as:

(33) $$\rho_{water}=\rho_{2}\cdot V_{f2}.$$

We performed the integration of Eq. 33 on both the entire domain and only on the inner region (which was divided into smaller mesh elements since affected by the interface motion, see Fig. 5), as well as on the outer region. The relative errors E were computed using the initial value as a reference. All data were exported from COMSOL® as text files, and re-processed with MATLAB® [46].

**Fig. 6:**
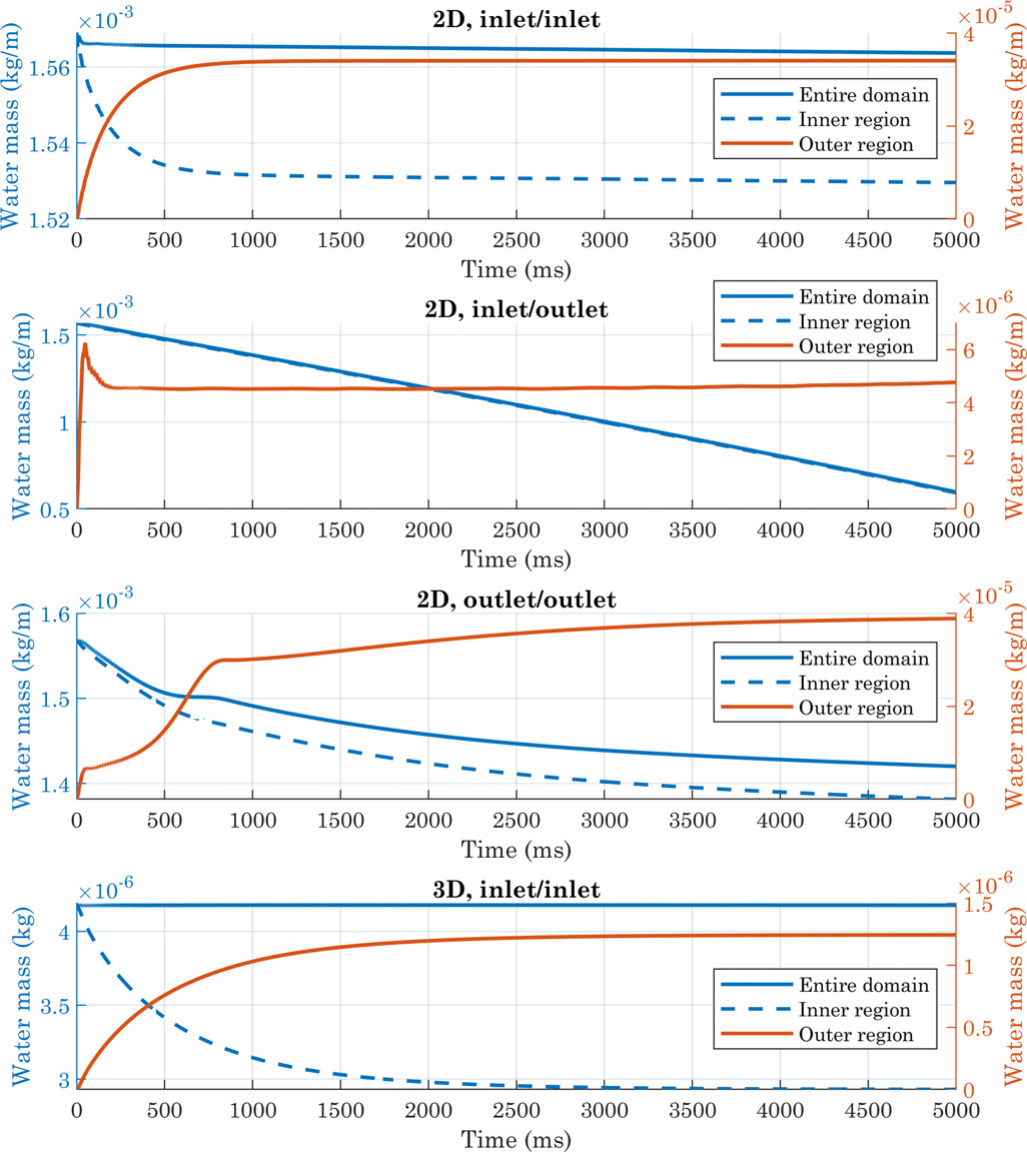
Comparison of deviations from mass conservation (absolute values) obtained with different boundary conditions applied to the upper, right, and left edges in simulations involving perfectly smooth surfaces. In the 2D cases, the volume of water is half of a cylinder of unit length, so the mass is expressed in kg/m.

**Fig. 7:**
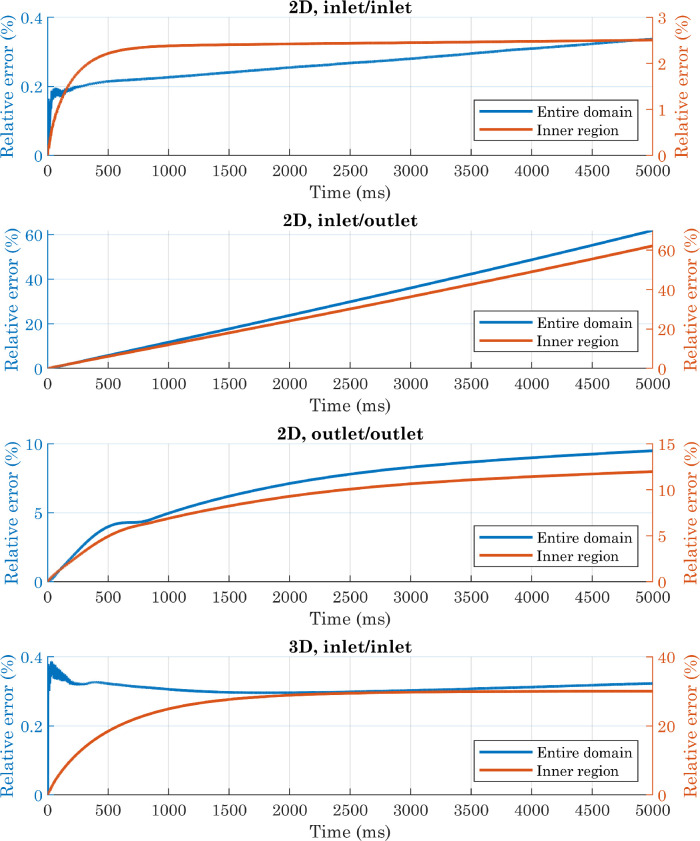
Comparison of deviations from mass conservation (relative errors) obtained with different boundary conditions applied to the upper, right, and left edges in simulations involving perfectly smooth surfaces. Relative errors were computed using the initial value as a reference.

From Figs. 6 and 7 it can be seen that the presence of an outlet condition greatly increases the mass change, which instead is kept low in the other two cases (when considering the entire domain). Comparing the “2D,inlet/inlet” and “3D,inlet/inlet” situations, it is clear, however, that for both of them, a portion of the calculated mass comes from the outer region, where theoretically no water should be present. This is due to the fact that the volume fraction of fluid two is not exactly zero in this area, for presumably numerical reasons. Although the absolute value of the integral evaluated on the outer region is lower in 3D simulations than in 2D simulations, the total volume of water at the beginning of the process is also lower in the former case: this should not be surprising, since we are comparing a sphere with a cylinder of equal radius but unit length. Overall, therefore, the lowest relative error occurs in the first of the situations analyzed. At the beginning of simulations, the difference between the integral evaluated only over the inner region and that calculated over the entire domain is negligible, because of the initial conditions imposed on the phase variable. The estimated mass in the inner region then decreases, eventually settling on a stable value in both the first and last configurations. In light of these considerations, subsequent simulations were performed on a two-dimensional computational domain, with inlet-type conditions.

The first simulations were carried out with smooth surfaces to test the model and calibrate its parameters (see Section 8). Next, several trapezoidal micro-structures with different geometric features were added at the solid wall (see Fig. 4).

## Simulation post-processing

The purpose of this protocol is to predict the wettability of micro-structured surfaces by means of continuum simulations, to facilitate the creation of systems with the desired hydrophilicity/hydrophobicity while reducing the time and cost of the design phase. The wetting properties of a surface are influenced by several factors: a hydrophobic surface, for example, may have excellent self-cleaning capabilities (lotus effect), or high adhesion (rose petal effect) [47], [48], [49]. In this study, we chose to characterize different surfaces by using the apparent contact angle, while simultaneously paying attention to which wetting state was achieved.

**Fig. 8:**
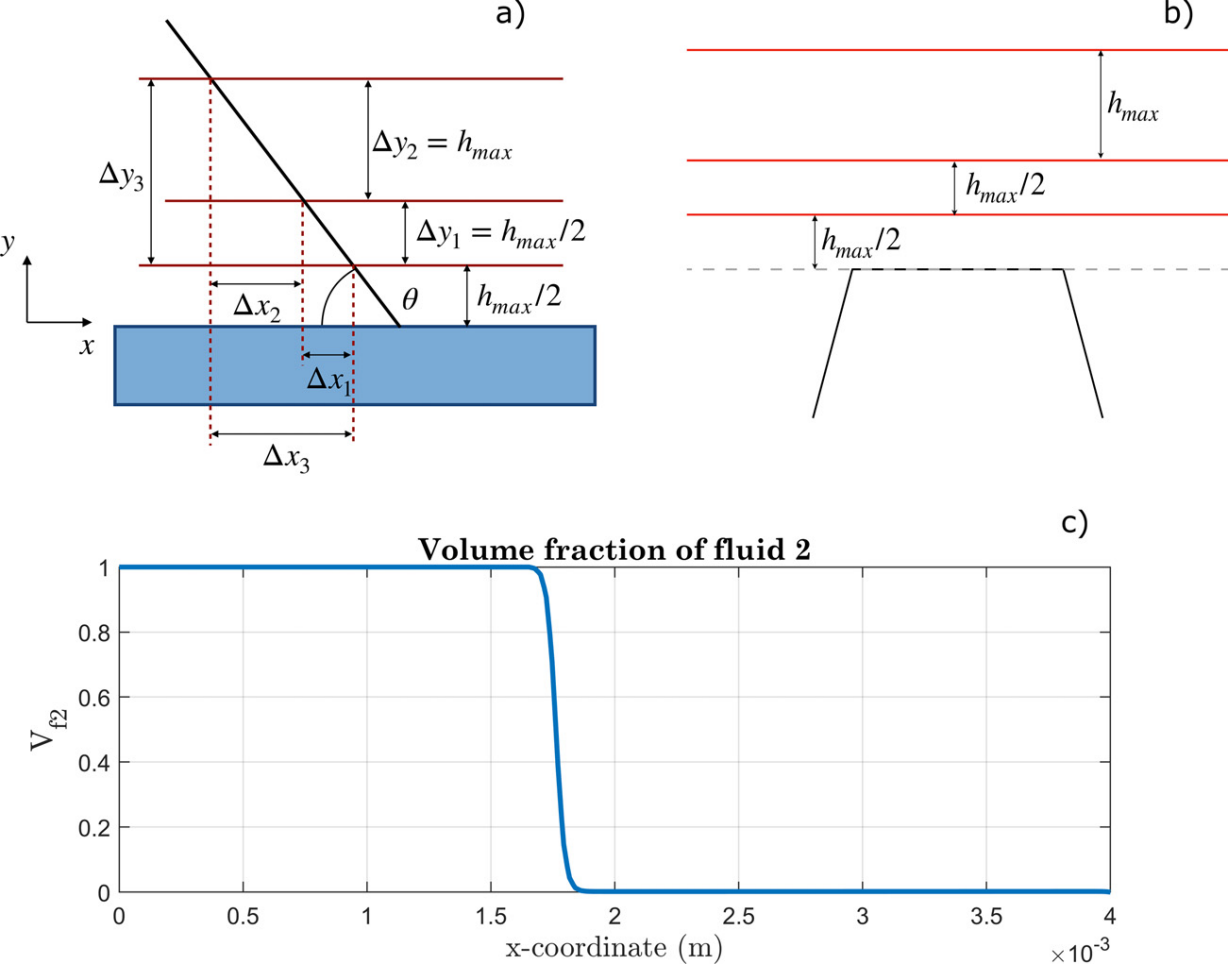
Evaluation of the contact angle using appropriately selected parallel lines in the case of (a) a smooth surface and (b) a micro-structured surface. (c) Example of a $V_{f2}$ profile along a horizontal line.

The apparent contact angle was evaluated at some distance from the solid wall, to avoid any local effect due to the imposed boundary conditions. Given a generic curve and a secant intersecting the curve at two distinct points A and B, the tangent is the line to which the secant tends when A and B are infinitely close to each other. We chose to exploit this definition for the evaluation of the final contact angle in our simulations, by approximating the water-air interface near the TPL with a straight line passing through two sufficiently close points. The procedure used is as follows:

• We selected three parallel lines, depicted in Fig. 8, and we extracted the profile of $V_{f2}$ along these lines at the end of the simulation.

**Fig. 9:**
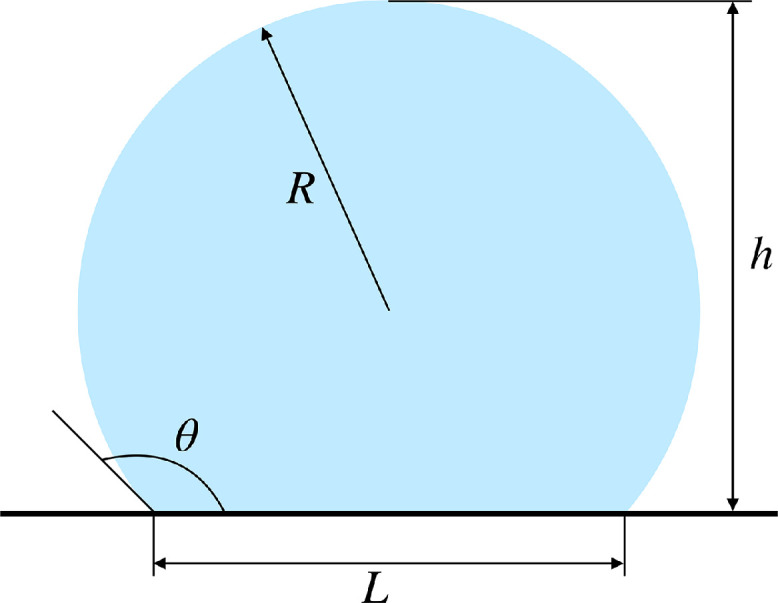
Geometric description of a droplet on a perfectly smooth surface, provided it takes the shape of a spherical cap.
• For each of the three extracted profiles, we identified the x coordinate corresponding to $V_{f2}=0.5$ (i.e., ϕ=0, the value representing the interface), by linear interpolation. We will call these positions $x_{1}$, $x_{2}$, and $x_{3}$ below, with reference to the three lines (from bottom to top).
• With the positions thus obtained, corresponding to the x coordinates of the intersections between the lines and the interface, the angles formed by the secants (passing through these points) and the horizontal direction were estimated. Since the distance between the parallel lines is sufficiently small, the secants can be considered a good approximation of the respective tangents. Defining $\Delta x_{1}=x_{1}-x_{2}$, $\Delta x_{2}=x_{2}-x_{3}$ and $\Delta x_{3}=x_{1}-x_{3}$, we computed:
  (34) $$\begin{array}{c} CA_{1}=\arctan\frac{0.5h_{max}}{\Delta x_{1}}, \end{array}$$
  (35) $$\begin{array}{c} CA_{2}=\arctan\frac{h_{max}}{\Delta x_{2}}, \end{array}$$
  (36) $$\begin{array}{c} CA_{3}=\arctan\frac{1.5h_{max}}{\Delta x_{3}}, \end{array}$$
  and thus $CA=\frac{CA_{1}+CA_{2}+CA_{3}}{3}$. If Δx<0, $CA=\pi -\arctan\frac{\Delta y}{\left|\Delta x\right|}$.

The values thus obtained (Table 1) were compared with the results of a post-processing performed with the “ImageJ” image analysis software [50]. This comparison showed substantial consistency between the software results and the contact angles evaluated with the procedure just explained. We chose to use the numerical method to estimate contact angles in subsequent simulations, to automate the procedure and reduce the subjectivity of processing as much as possible. We repeated the same procedure by reducing the mesh size to check that the result was not significantly affected by the interface thickness. In a phase-field simulation, as a matter of fact, the outcome is physically reliable only when it is not meaningfully influenced by the numerical thickness chosen for the interface, since the widths of interfaces in real systems are much smaller than those used in simulations. As shown by the results in Table 1, simulations performed with perfectly smooth surfaces led to values in agreement with the imposed boundary conditions and not significantly affected by variations in interface thickness. These outcomes were also found to be consistent with geometric evaluations commonly used to describe a droplet in equilibrium on a perfectly smooth surface, barring minor deviations also mentioned in the next section (see Table 1, (37) and Reference [51]).

**Table 1:** Results of simulations carried out with smooth surfaces, imposing a contact angle on the wall ($CA_{w}$) of 70${}^{\circ }$. Comparison between different methods used to evaluate the CA. The designation “$E_{tot}$” refers to the percentage error related to water mass conservation (estimated over the entire domain). This definition will also be used in subsequent tables. $l_{g}$ and $h_{g}$ were evaluated from geometric considerations using the contact angle defined through the boundary condition, i.e., $70^{\circ }$. l and h, on the other hand, were obtained from simulations. As can be seen, the two ratios ($l_{g}/h_{g}$ and l/h) differ by about 4%. For further details, please refer to Fig. 9 and Reference [51].

ϵ (μm)	$CA_{w}$ (${}^{\circ }$)	$CA_{1}$ (${}^{\circ }$)	$CA_{2}$ (${}^{\circ }$)	$CA_{3}$ (${}^{\circ }$)	CA (${}^{\circ }$)	$CA_{ImJ}$ (${}^{\circ }$)	$E_{tot}\;\left(\%\right)$	l/h	$l_{g}/h_{g}$
23.5	70	70.33	70.69	70.57	70.53	69±2	0.22	1.48	1.43
11.8	70	70.75	71.52	71.26	71.18	70±2	0.12	1.49	1.43

Gravity can significantly affect the resting shape of liquid droplets, especially large ones. In the simulations performed, the equations include the gravitational effect, although droplet sizes smaller than the capillary length were considered. The gravitational effect, along with micro-structures added to the geometry as well as numerical approximations, could result in small deviations of the droplet profile from the spherical shape. If the drop was perfectly comparable to a spherical cap, indeed, the contact angle could be evaluated as [52]:

(37) $$\theta =\left\{ \begin{array}{c} \pi -\arcsin(\frac{2lh}{l^{2}+h^{2}}),\;\;\;\text{if}\;h>R\\ \arcsin(\frac{2lh}{l^{2}+h^{2}}),\;\;\;\text{if}\;h\leq R \end{array}\right.$$

where $l=\frac{L}{2}$ and $R=\frac{l^{2}+h^{2}}{2h}$ (Fig. 9). Comparing the results provided by these expressions with the contact angles evaluated with the tangent method shows differences of a few degrees between the two approaches, ascribable to deviations of the shape from the spherical one and the approximations made. Table 2 shows the results for the two approaches in the case of simulations carried out with a micro-structured surface.

**Table 2:** Results of simulations carried out with micro-structured surfaces (A=24μm,B=80μm,D=120μm,H=200μm, ϵ=15.7μm, see Fig. 4). Comparison of contact angle values obtained from the geometric definition of spherical cap ($CA_{geom}$) and contact angles evaluated using the tangent definition ($CA_{1}$ and CA, see (34), (35), (36)).

$CA_{w}\;\left({}^{\circ }\right)$	$CA_{1}\;\left({}^{\circ }\right)$	$CA\;({}^{\circ })$	$CA_{geom}\;\left({}^{\circ }\right)$
120	159.1	159.8	153.8
110	152.5	151.9	145.4
105	151.8	151.0	144.9
100	135.9	134.1	128.2
95	134.1	133.1	128.0
85	125.7	124.6	119.3
80	125.0	124.3	119.1
75	116.5	115.0	110.5
70	116.0	114.8	110.4
60	99.3	98.1	93.2
50	91.0	89.8	84.6
30	66.4	62.9	55.8

**Fig. 10:**
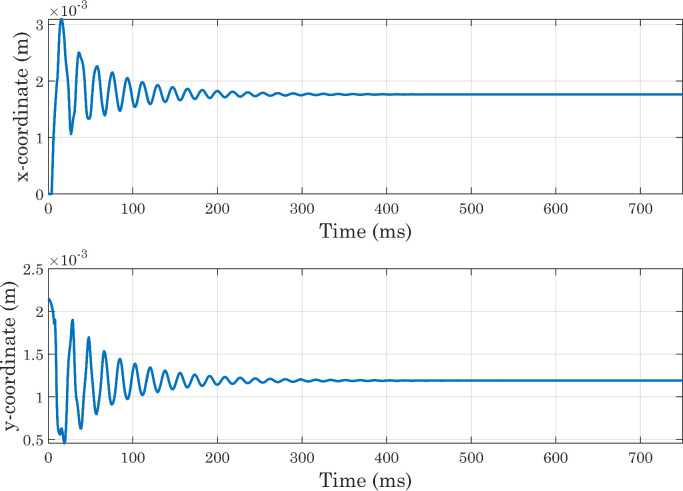
Analysis of simulations performed with a perfectly smooth surface: evolution of the interface positions over time ($CA_{w}=70^{\circ }$, $h_{\max}=2.35\times 10^{-5}\;m$, χ=100m·s/kg). $CA_{w}$ is the angle imposed at the wall, and $h_{max}$ is the maximum size selected for the mesh elements in the area of the domain crossed by the interface motion (inner region). These definitions will also be used in subsequent images.

**Fig. 11:**
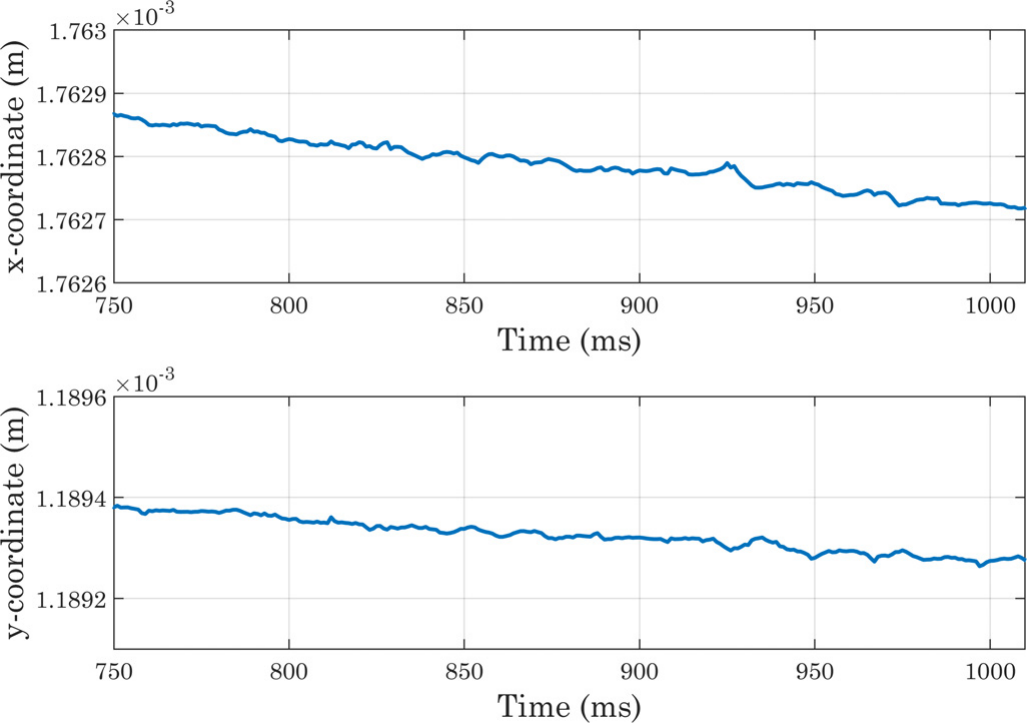
Analysis of simulations performed with a perfectly smooth surface: evolution of the interface positions over time, magnified view ($CA_{w}=70^{\circ }$, $h_{\max}=2.35\times 10^{-5}\;m$, χ=100m·s/kg).

**Fig. 12:**
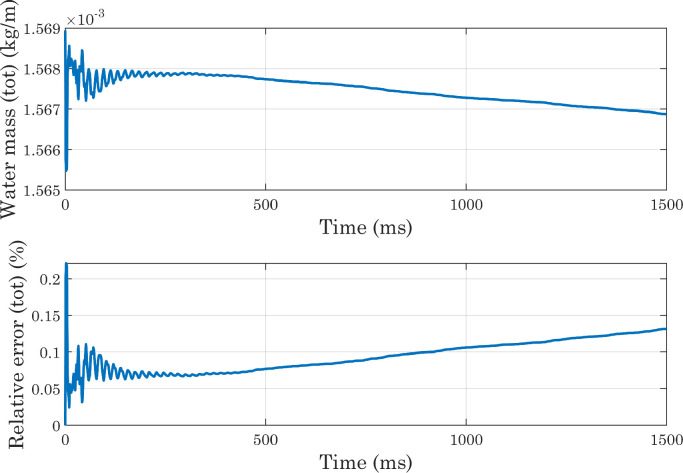
Analysis of simulations performed with a perfectly smooth surface: water mass conservation, calculated from the domain-wide integration ($CA_{w}=70^{\circ }$, $h_{\max}=2.35\times 10^{-5}\;m$, χ=100m·s/kg).

**Fig. 13:**
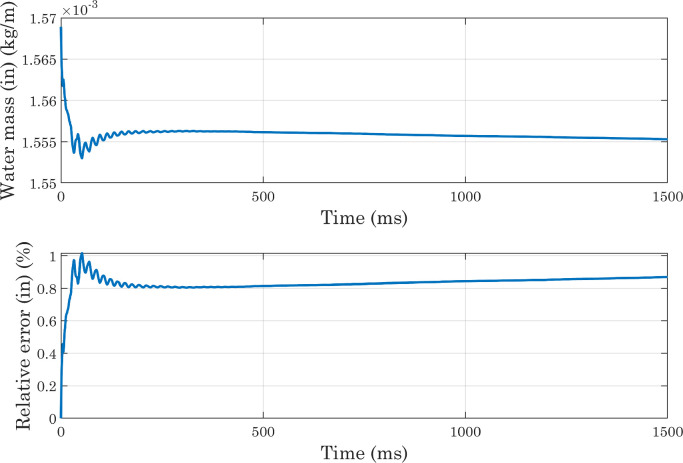
Analysis of simulations performed with a perfectly smooth surface: water mass conservation, with integration carried out on the inner region of the domain ($CA_{w}=70^{\circ }$, $h_{\max}=2.35\times 10^{-5}\;m$, χ=100m·s/kg).

**Fig. 14:**
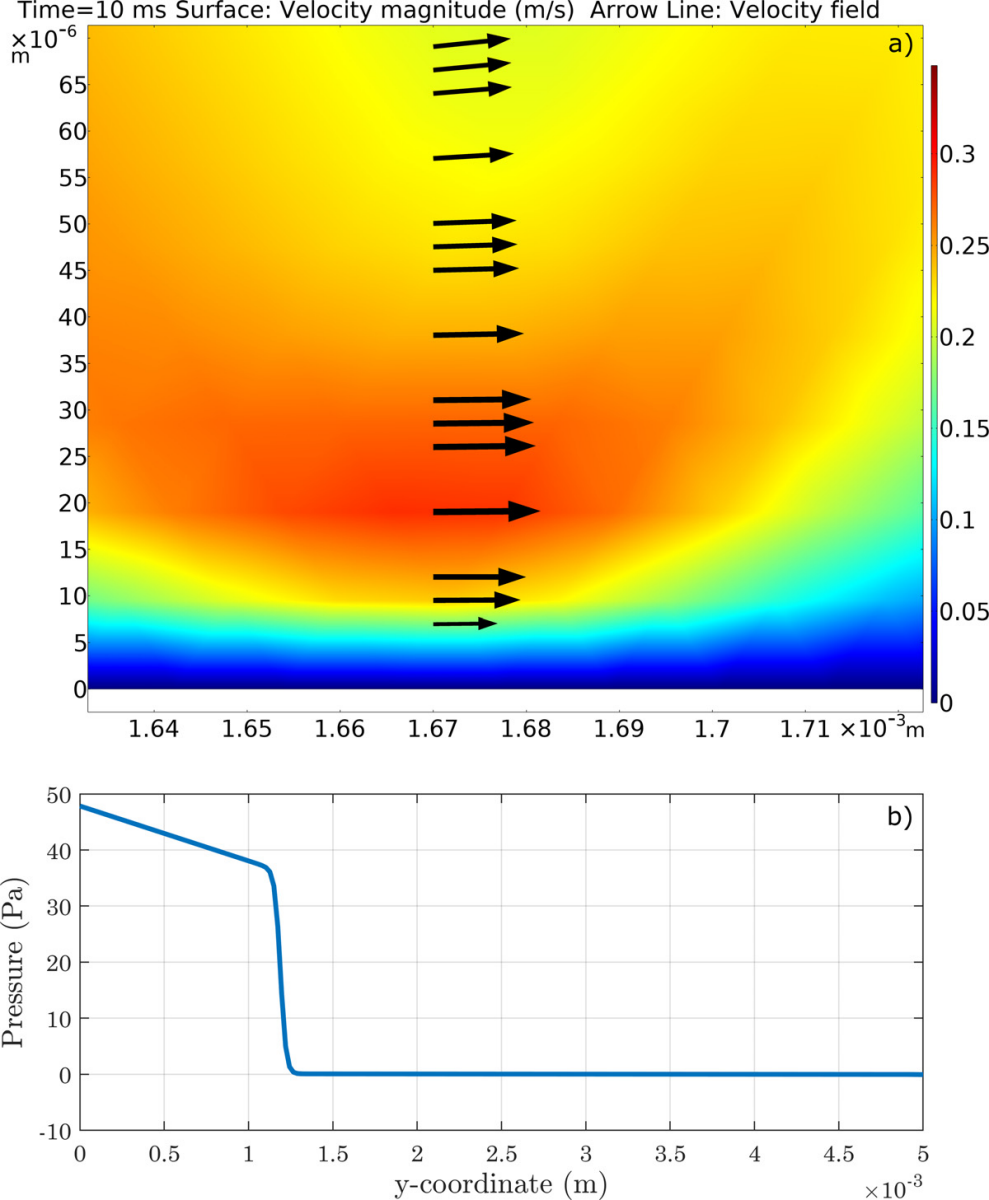
Analysis of simulations carried out with a perfectly smooth surface: verification of compliance with the boundary conditions imposed on pressure and velocity. (a) Velocity profile near the solid wall during the movement of the TPL. (b) Relative pressure profile along the y axis. The velocity near the solid wall is actually zero, although the diffusion introduced by the Cahn-Hilliard model makes contact line motion possible. As for pressure, this is essentially equal to the atmospheric value throughout the domain, except inside the droplet.

**Fig. 15:**
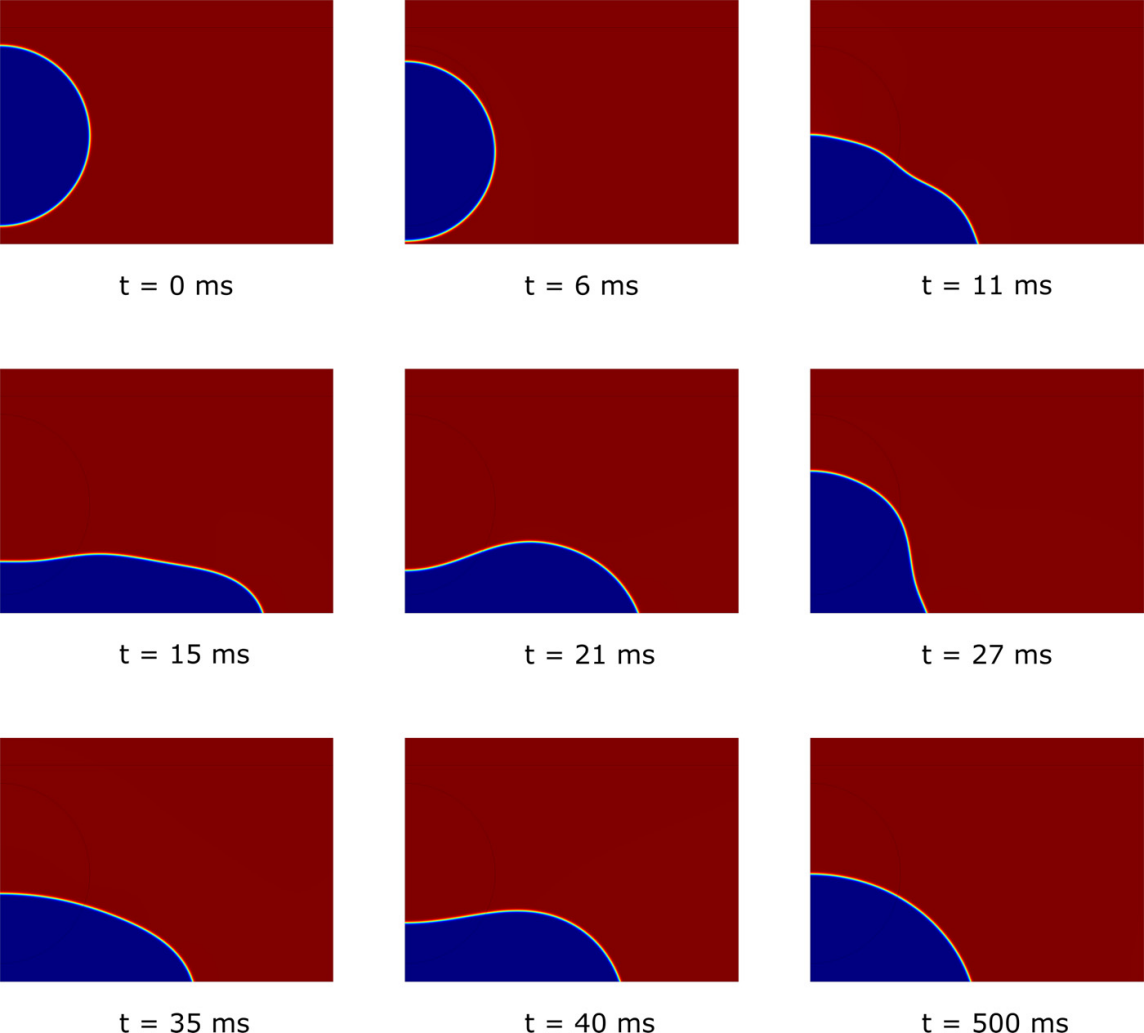
Representation of the collision, spreading, and rebound process of a droplet on a perfectly smooth surface, simulated using the phase-field model ($CA_{w}=70^{\circ }$). Different colors identify the fraction of air in the domain: blue corresponds to $V_{f1}=0$ (ϕ=1), red to $V_{f1}=1$ (ϕ=−1, see Fig. 4).

As the main purpose of this protocol is to analyze not the dynamics of the droplet spreading, but the equilibrium condition reached at the end of the process, it is also necessary to verify that the state attained by the drop at the end of simulations is sufficiently stable. For this reason, we decided to extract the $V_{f2}$ profile at each time-step along a horizontal line placed at a certain distance from the x axis, and to identify for each of them the x coordinate corresponding to the interface position. The same procedure was applied along the y axis. From Fig. 10 it can be seen that the positions thus identified follow oscillatory trends and then settle on stable values, although small movements are always present (as shown in Fig. 11) for presumably numerical reasons. In each simulation, the conservation of water mass was always verified [36]. For the sake of brevity, only the graphs for the first of the simulations shown in Table 1 are presented here (Figs. 12 and 13). As a final check on the consistency of the model, we verified that the boundary conditions imposed on pressure and velocity were respected (Fig. 14). All these tests were performed in simulations with both smooth and micro-structured surfaces. Finally, some images illustrating the process of impact, spreading, and rebound of the droplet on a smooth surface are provided (Fig. 15).

## Calibration of phase-field model parameters

The most interesting parameters for what concerns the adopted model Eqs. 16 and (17) are the interface thickness ϵ and the mobility tuning parameter χ, which are the degrees of freedom of the selected phase-field model.

**Fig. 16:**
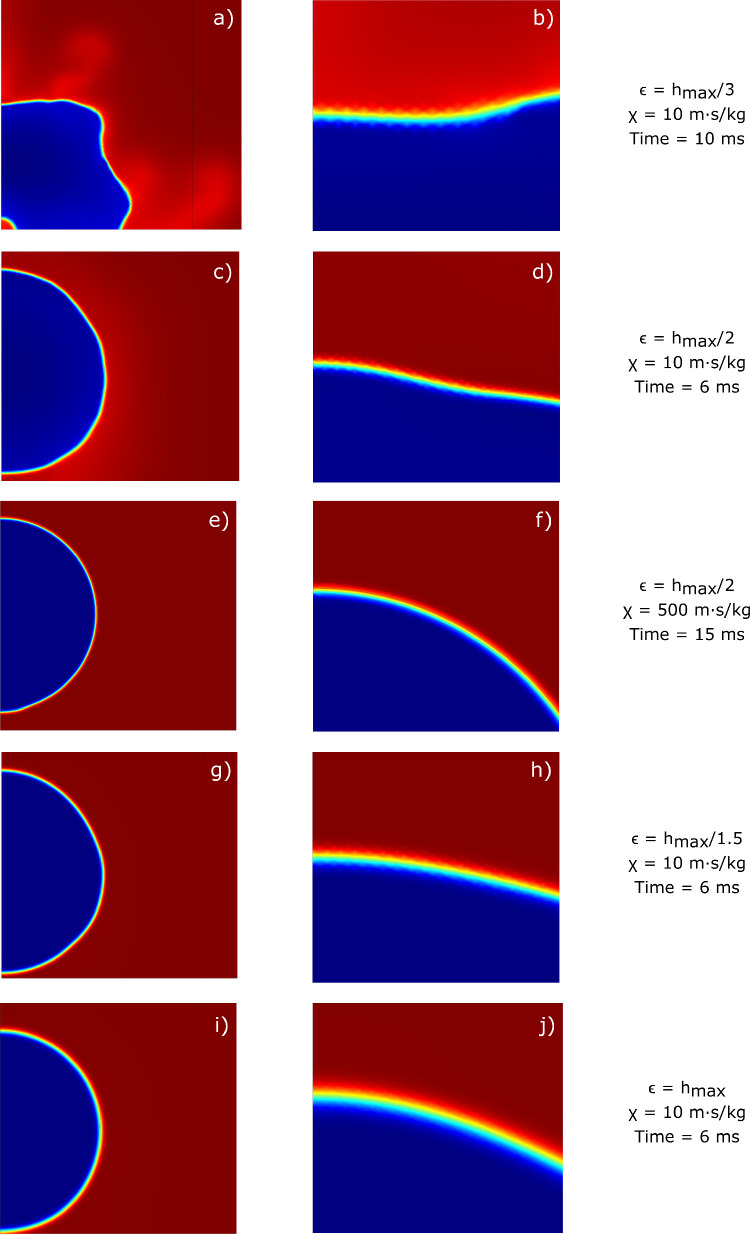
Examples of interface deformation with different values of interface thickness and mobility parameters. Different colors identify the fraction of air in the domain: blue corresponds to $V_{f1}=0$ (ϕ=1), red to $V_{f1}=1$ (ϕ=−1, see Fig. 4). (a) $\epsilon =h_{max}/3$, χ=10m·s/kg, Time = 10ms; (b) magnified view of the interface. (c) $\epsilon =h_{max}/2$, χ=10m·s/kg, Time = 6ms; (d) magnified view of the interface. (e) $\epsilon =h_{max}/2$, χ=500m·s/kg, Time = 15ms; (f) magnified view of the interface. (g) $\epsilon =h_{max}/1.5$, χ=10m·s/kg, Time = 6ms; (h) magnified view of the interface. (i) $\epsilon =h_{max}$, χ=10m·s/kg, Time = 6ms; (j) magnified view of the interface.

With regard to the thickness of the interface, many authors suggest using a width equal to or greater than half of the maximum mesh size [35], [53]. Therefore, we chose to compare the results obtained with different ϵ values to select the most appropriate one. A fixed, non-uniform, triangular mesh was selected for this study, and a finer grid was chosen for the area affected by the movement of the interface (inner region): $h_{max}$ is the value of the maximum mesh element size selected in the software for the area with the finer grid. From Fig. 16, we can notice that when ϵ is smaller than half of the mesh size, the deformation of the interface is abnormal: its configuration is not consistent with the physics of the process, and a jagged profile and non-constant thickness can be seen from the magnification. Moreover, the diffusive phenomenon due to the Cahn-Hilliard equations occurs incorrectly, as evidenced by the presence of regions distant from the interface where the volume fraction takes on values far from those for pure components (ϕ=±1). A similar, though less obvious, situation can be observed when the set interface thickness is exactly half of the mesh size configured in the software. Since the diffusion of the phase-field model is governed by the mobility parameter M (which, in turn, is related to the interface thickness and the mobility tuning parameter), we tried to assess whether the incorrect deformation of the interface could be attributed to the low mobility by increasing χ. However, from Fig. 16 we can see that, although the interface no longer exhibits the jagged appearance identified in previous cases, the system fails to evolve, and the drop remains in a position close to its initial location. The problem, therefore, probably lies in the magnitude of ϵ with respect to the set value of $h_{max}$: to further support this hypothesis, the interface shows a nearly constant thickness and smoother profile when increasing the interface thickness up to the mesh size, and the evolution of the separation surface appears to be physically consistent. Given the above evidence, we chose to use an interface thickness $\epsilon =h_{max}$.

**Fig. 17:**
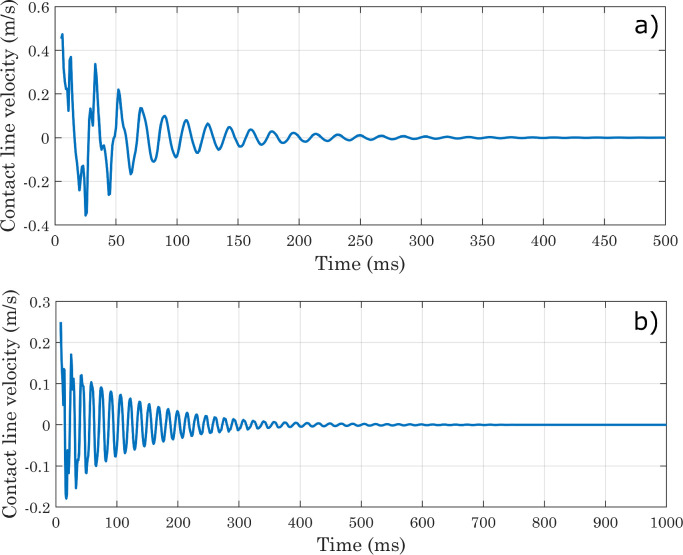
Estimation of the contact line velocity with respect to the solid (perfectly smooth) wall, by numerically deriving the x coordinates of the interface as a function of time. (a) $CA_{w}=70^{\circ }$; (b) $CA_{w}=120^{\circ }$.

As far as the mobility parameter is concerned, several approaches have been proposed in the literature to guide its definition, often based on the need to obtain phase-field simulations that do not depend on the interface thickness (convergence to the sharp-interface limit). If the interface does not intersect a solid wall, this convergence value exists and is unique, and it can be achieved for $Cn=O(10^{-2})$ ($Cn=\frac{\epsilon }{D}$ is the Cahn number, i.e. the ratio of interface thickness to characteristic length). Conversely, when the separating surface intersects a solid wall, the diffusion produces the displacement of the TPL, and achieving convergence becomes a non-trivial problem. Yue et al. [2] verified the existence of the sharp-interface limit for these cases, and developed a practical criterion for choosing M to approach convergence with a finite value of Cn. The criterion has been applied many times over the years, and proposes $Cn\leq 4\frac{\sqrt{M\mu }}{D}$ as a limit for achieving convergence by reducing Cn while keeping the other parameters fixed. This relation, however, allows only the lower limit of M (and consequently the minimum value of χ) to be determined, while the appropriate value for a given ϵ can be found by comparison with experimental data: different mobility parameters lead to dissimilar results [35]. Moreover, Yue et al. [2] analyzed a Couette flow involving two fluids of comparable viscosity, for which a power law scaling $M∼\epsilon^{0}$ was found. To account for situations where the viscosities are highly dissimilar, the use of an effective μ value calculated as a geometric mean was proposed: $\mu_{e}=\sqrt{\mu_{1}\mu_{2}}$, where $\mu_{1}$ and $\mu_{2}$ are the viscosities of the two fluids involved. Magaletti et al. also emphasized the importance of defining an asymptotic state where the macroscopic behavior is independent of the specific values of ϵ and M used [33], pointing out that mobility should suitably change as the interface thickness decreases. They proposed the following scaling law to assess the optimal mobility: $M_{opt}^{*}≃3Cn^{2}$, where $M^{*}=\frac{3M\sigma }{2\sqrt{2}D^{3}\omega }$, U=ωD is the characteristic velocity, ω the frequency. A similar expression is suggested by the COMSOL^Ⓡ^ user’s guide [53]. More recently, Demont et al. [54] investigated the open questions concerning the optimal scaling of the mobility parameter M and the approach to the sharp-interface limit solution for an oscillating droplet, finding that the deviation between the diffuse-interface solution and its sharp-interface limit decreases according to O(ϵ) when the interface thickness (ϵ) tends to zero. Similarly, Schmeller and Peschka [55] focused their attention on conducting a systematic examination of the optimal selection of the Cahn-Hilliard mobility. In particular, they showed that there are optimal values for M(ϵ) for each ϵ, and this optimal mobility decreases for smaller ϵ.

**Fig. 18:**
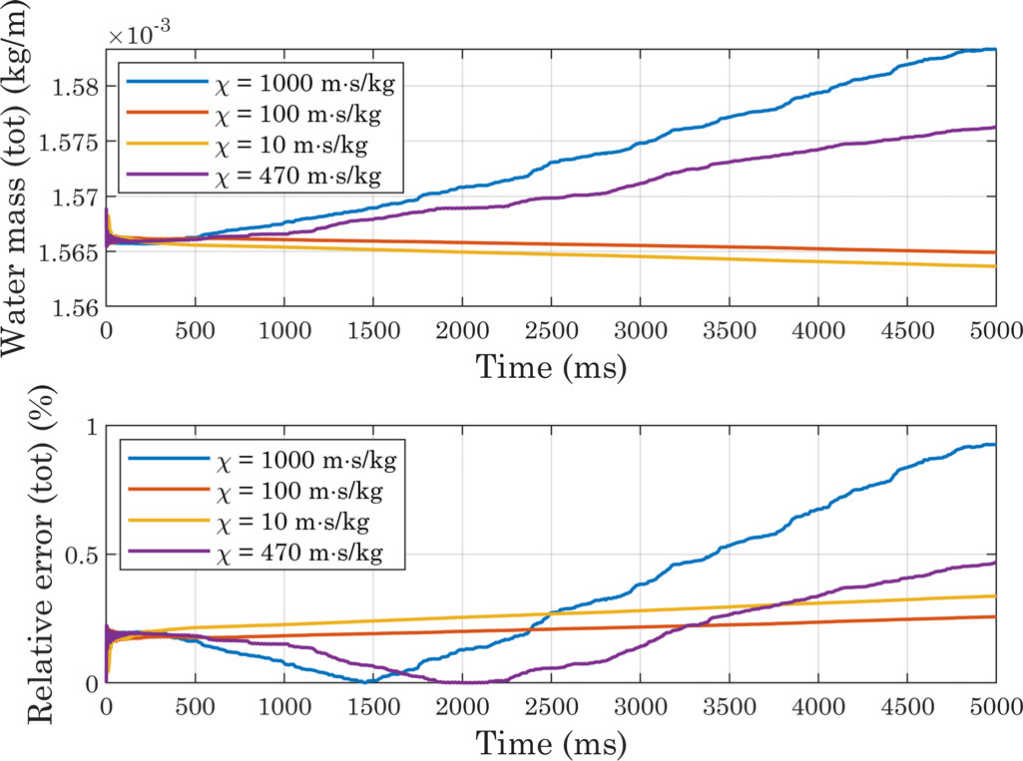
Conservation of water mass in simulations carried out with perfectly smooth surfaces while varying the mobility parameter ($CA_{w}=120^{\circ }$, $h_{\max}=2.35\times 10^{-5}\;m$). For interpretation of the color references in the legend of this figure and subsequent ones, the reader is referred to the web version of this article.

**Fig. 19:**
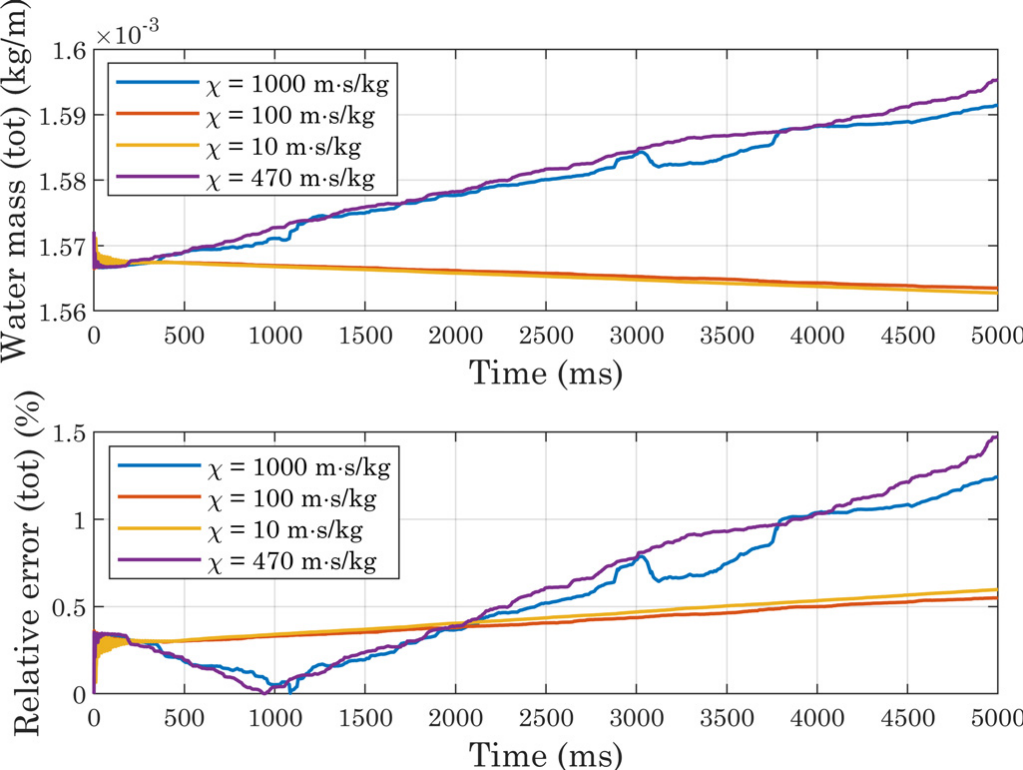
Conservation of water mass in simulations carried out with perfectly smooth surfaces while varying the mobility parameter ($CA_{w}=120^{\circ }$, $h_{\max}=4.7\times 10^{-5}\;m$).

**Fig. 20:**
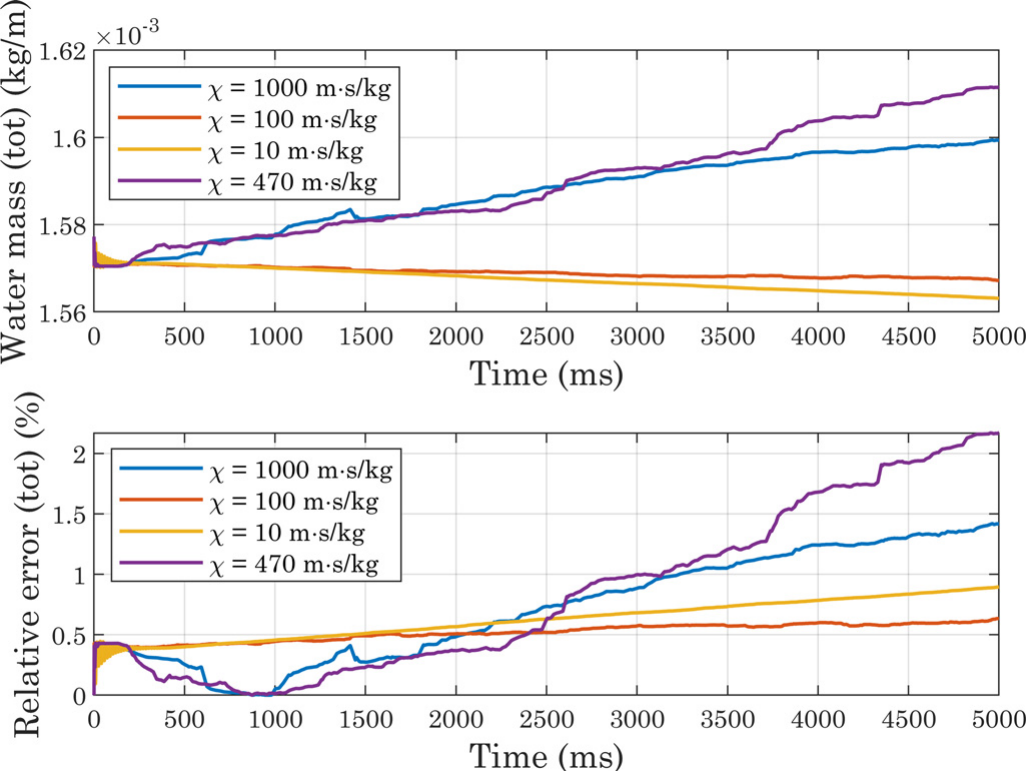
Conservation of water mass in simulations carried out with perfectly smooth surfaces while varying the mobility parameter ($CA_{w}=120^{\circ }$, $h_{\max}=7.05\times 10^{-5}\;m$).

**Fig. 21:**
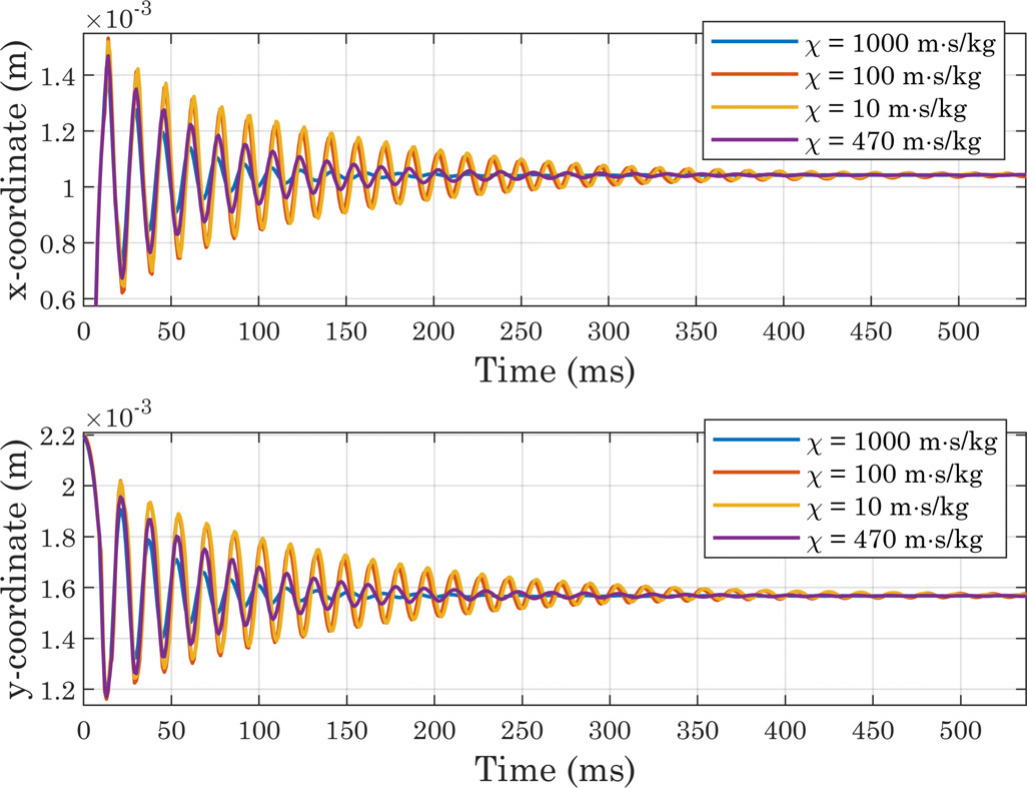
Evolution of the interface positions in simulations carried out with perfectly smooth surfaces while varying the mobility parameter ($CA_{w}=120^{\circ }$, $h_{\max}=2.35\times 10^{-5}\;m$).

**Fig. 22:**
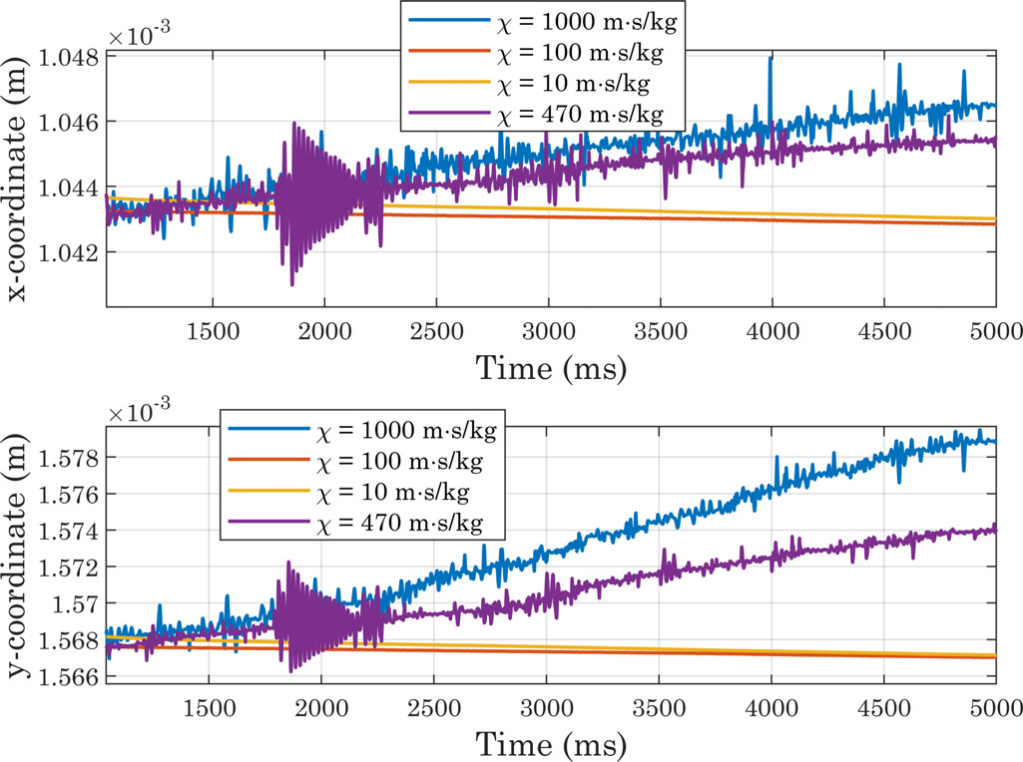
Evolution of the interface positions (magnified view) in simulations carried out with perfectly smooth surfaces while varying the mobility parameter ($CA_{w}=120^{\circ }$, $h_{\max}=2.35\times 10^{-5}\;m$).

**Fig. 23:**
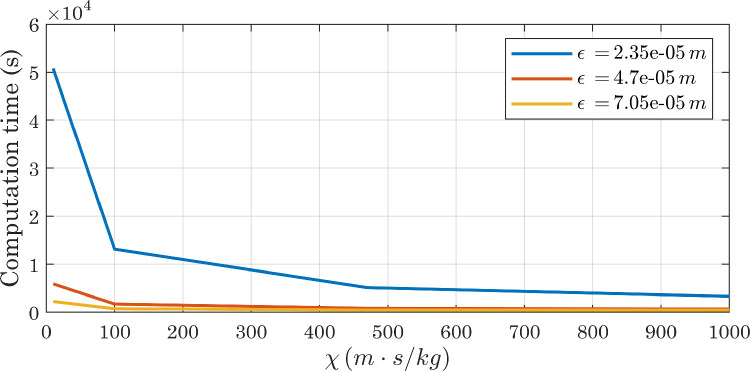
Computational cost of simulations with perfectly smooth surfaces as the mobility parameter and mesh size change ($CA_{w}=120^{\circ }$).

Table 3 shows the results provided by the different approaches, together with some dimensionless numbers. Given the conflicting results provided by the analytical formulations (see Table 3), we decided to treat mobility, and in particular the tuning parameter χ and the interface thickness ϵ, as phenomenological parameters. We ran several simulations, setting a contact angle at the wall of $CA_{w}=120^{\circ }$ and systematically changing the mesh size $h_{max}$ (hence the interface thickness ϵ) and the parameter χ. All simulations resulted in final contact angles consistent with each other and with the imposed boundary conditions [36]. A comparison of Figs. 18, 19 and 20 shows that by increasing the mesh size while keeping χ fixed, the error associated with water mass conservation increases: the lowest mass variations are observed with χ=100m·s/kg, whereas for high values of the tuning parameter markedly increasing trends are visible. All simulations reached a sufficiently stable state, but increasing mobility dampens the oscillation amplitude more quickly, and at the same time changes their phase (Fig. 21). The substantial instability associated with higher values of χ, already evident in the mass evaluation, can also be observed by analyzing the interface positions: in the magnified views presented in Fig. 22, the slight oscillations already mentioned above become more pronounced at χ=470m·s/kg and χ=1000m·s/kg. Also in view of the computational costs, shown in Fig. 23, we considered it appropriate to use χ=100m·s/kg as a first guess in subsequent simulations.

**Table 3:** Dimensionless numbers and mobility tuning parameter, estimated with different approaches. We chose to use the droplet diameter before impact as the characteristic length D[27] and to assume the contact line velocity as U[3]: after running the simulations, we estimated the actual contact line speed with respect to the solid wall, by numerically deriving the x coordinate as a function of time (see Fig. 17).

	Water	Air	Effective
μ(Pa·s)	$1.002\times 10^{-3}$	$1.81\times 10^{-5}$	$1.35\times 10^{-4}$
$\rho \;(\mathrm{kg}/m^{3})$	998	1.205	34.68
ϵ(m)	$2.35\times 10^{-5}$	-	$2.35\times 10^{-5}$
D(m)	0.002	-	0.002
σ(N/m)	0.073	-	0.073
U(m/s)	0.2	-	0.2
ω(Hz)	100	-	100
Cn	0.012	-	0.012
Re	398	-	103
Ca	0.003	-	0.0004
We	1.09	-	0.038
$Fr^{2}$	2.04	-	2.04
$\chi_{\mathrm{Yue}}\;\left(m\cdot s/\mathrm{kg}\right)$	62	-	464
$\chi_{\mathrm{Maga}\mathrm{letti}}\;\left(m\cdot s/\mathrm{kg}\right)$	7.75	-	7.75

With the introduction of micro-structures, however, achieving a ϵ-independent solution (i.e., convergence to the sharp-interface limit) may not be obvious, because interactions between the interface and geometric elements with length scales comparable to its thickness can occur. For this reason, in simulations with micro-structured surfaces, we varied the thickness of the interface until we achieved a result that was essentially independent of the chosen ϵ-value (see Tables 4, 5 and 6 and reference [36] for further details). We also performed some simulations to assess the influence of mobility, geometry, and contact angle on the convergence limit (Tables 7, 8, 9 and 10). For all simulations, conservation of water mass and achievement of a sufficiently stable state were verified [36].

**Table 4:** Results of simulations performed with micro-structured surfaces: analysis of the dependence of the result on the considered interface thickness (A=24μm,B=80μm,D=120μm,H=200μm).

ϵ(μm)	$CA_{w}\;\left({}^{\circ }\right)$	$CA_{1}\;\left({}^{\circ }\right)$	$CA\;({}^{\circ })$	$E_{tot}\;\left(\%\right)$
94	70	35.3	33.3	0.71
47	70	65.5	63.8	0.38
35.3	70	83.9	80.4	0.30
23.5	70	102.0	98.6	0.22
15.7	70	116.0	114.8	0.17
13.4	70	116.0	115.1	0.14
11.8	70	115.4	115.2	0.12
10	70	116.0	115.4	0.11

**Table 5:** Results of simulations performed with micro-structured surfaces: analysis of the dependence of the result on the considered interface thickness (A=12μm,B=40μm,D=60μm,H=100μm). Since we divided each dimension of the micro-structure by two compared to the previous case, the maximum suitable interface thickness is also half of the former.

ϵ(μm)	$CA_{w}\;\left({}^{\circ }\right)$	$CA_{1}\;\left({}^{\circ }\right)$	$CA\;({}^{\circ })$	$E_{tot}\;\left(\%\right)$
47	70	31.9	30.4	0.42
35.3	70	43.5	43.1	0.30
23.5	70	69.1	67.7	0.22
15.7	70	94.3	91.5	0.16
11.8	70	105.6	103.7	0.12
7.8	70	116.7	115.9	0.08
6.7	70	116.2	115.6	0.07

**Table 6:** Results of simulations performed with micro-structured surfaces: analysis of the dependence of the result on the considered interface thickness (A=6μm,B=20μm,D=30μm,H=50μm). With these dimensions, convergence could not be achieved for any of the thicknesses used.

ϵ(μm)	$CA_{w}\;\left({}^{\circ }\right)$	$CA_{1}\;\left({}^{\circ }\right)$	$CA\;({}^{\circ })$	$E_{tot}\;\left(\%\right)$
47	70	48.6	46.7	0.63
35.3	70	26.5	24.6	0.30
23.5	70	31.2	29.9	0.22
15.7	70	51.5	51.2	0.13
11.8	70	71.0	69.7	0.11

**Table 7:** Results of simulations performed with micro-structured surfaces: analysis of the effect of mobility tuning parameter on convergence (A=24μm,B=80μm,D=120μm,H=200μm). The mobility tuning parameter also plays an important role in defining the suitable mesh size: with the same geometry, as χ decreases, the interface thickness ϵ must also be reduced to achieve convergence.

χ(m·s/kg)	ϵ(μm)	$CA_{w}\;\left({}^{\circ }\right)$	$CA_{1}\;\left({}^{\circ }\right)$	$CA\;({}^{\circ })$	$E_{tot}\;\left(\%\right)$
20	15.7	100	143.1	142.1	0.21
100	15.7	100	135.9	134.1	0.16
300	15.7	100	135.9	134.1	0.16
50	15.7	70	108.1	106.9	0.15
100	15.7	70	116.0	114.8	0.17
100	15.7	50	91.0	89.8	0.16
500	15.7	50	91.0	89.7	0.16
20	11.8	50	91.4	90.1	0.11
100	11.8	50	91.4	90.1	0.12

**Table 8:** Results of simulations performed with micro-structured surfaces: analysis of the effect of micro-structure geometry on convergence. The results analyzed so far have shown that the mesh size for which convergence is achieved is influenced by the characteristic dimensions of the micro-structure geometry. This table also reveals that, by changing just the height of the trapezoids, the convergence condition varies.

ϵ(μm)	H(μm)	A(μm)	B(μm)	D(μm)	$CA_{w}\;\left({}^{\circ }\right)$	$CA_{1}\;\left({}^{\circ }\right)$	$CA\;({}^{\circ })$	$E_{tot}\;\left(\%\right)$
15.7	100	24	80	120	70	107.7	107.6	0.16
11.8	100	24	80	120	70	117.7	117.4	0.12
9.4	100	24	80	120	70	118.1	117.2	0.10
15.7	300	24	80	120	70	114.4	113.1	0.17
11.8	300	24	80	120	70	113.5	112.9	0.12

**Table 9:** Results of simulations performed with micro-structured surfaces: analysis of the effect of the CA imposed at the solid wall on convergence (A=24μm,B=80μm,D=120μm,H=200μm). With both a contact angle of 120${}^{\circ }$ and 50${}^{\circ }$, varying the thickness of the interface does not significantly change the result.

ϵ(μm)	$CA_{w}\;\left({}^{\circ }\right)$	$CA_{1}\;\left({}^{\circ }\right)$	$CA\;({}^{\circ })$	$E_{tot}\;\left(\%\right)$
15.7	120	159.1	159.8	0.17
11.8	120	161.4	160.4	0.13
15.7	50	91.0	89.8	0.16
11.8	50	91.4	90.1	0.12

**Table 10:** Results of simulations performed with micro-structured surfaces. To further verify the stability of the model, we decided to run a simulation by translating the micro-structure slightly, to check that the exact point where the droplet impacts does not significantly alter the wetting state. (a) Original micro-structure; (b) shifted geometry. The behavior of the system appears to be essentially unchanged, despite the alteration introduced: in both cases, a Wenzel state was achieved, with a difference of a few degrees in the final contact angle.

$CA_{w}\;\left({}^{\circ }\right)$	A(μm)	B(μm)	D(μm)	H(μm)	ϵ(μm)	$CA_{1}\;\left({}^{\circ }\right)$	$CA\;({}^{\circ })$	$E_{tot}\;\left(\%\right)$
100	24	80	120	200	15.7	135.9	134.1	0.16
100	24	80	120	200	15.7	138.7	138.2	0.17

It is worth noting that the parameters deemed suitable within the scope of this study may not be adequate for simulating phenomena of a different nature. This work is intended to suggest some analyses and procedures to be considered in the parameter calibration process, which will hopefully be useful in the future. Therefore, for each simulated phenomenon it would be advisable to repeat the calibration process, also taking advantage of any experimental data that may be available. Moreover, some simplifying assumptions and approximations (such as the use of a 2D domain or a static boundary condition for the contact angle) were employed as considered appropriate in the context of this analysis, aimed at evaluating the static contact angle of a droplet on micro-structured surfaces. For future developments of this study, we are currently considering some improvements to increase the accuracy of the simulations, such as using a dynamic boundary condition for the contact angle.

## CRediT authorship contribution statement

**Marina Provenzano:** Methodology, Software, Validation, Formal analysis, Investigation, Data curation, Writing – original draft, Visualization. **Francesco Maria Bellussi:** Methodology, Software, Validation, Writing – original draft, Visualization. **Matteo Morciano:** Methodology, Software, Writing – original draft, Visualization. **Pietro Asinari:** Supervision, Project administration, Funding acquisition. **Matteo Fasano:** Conceptualization, Methodology, Resources, Writing – review & editing, Supervision.

## Declaration of Competing Interest

The authors declare that they have no known competing financial interests or personal relationships that could have appeared to influence the work reported in this paper.

## Data Availability

Data will be made available on request.
